# A multimodal dataset for various forms of distracted driving

**DOI:** 10.1038/sdata.2017.110

**Published:** 2017-08-15

**Authors:** Salah Taamneh, Panagiotis Tsiamyrtzis, Malcolm Dcosta, Pradeep Buddharaju, Ashik Khatri, Michael Manser, Thomas Ferris, Robert Wunderlich, Ioannis Pavlidis

**Affiliations:** 1 Computational Physiology Laboratory, University of Houston, Houston, Texas 77204, USA; 2 Department of Statistics, Athens University of Economics and Business, Athens 104 34, Greece; 3 Elizabeth City State University, Department of Mathematics and Computer Science, Elizabeth City, North Carolina 27909, USA; 4 Computer Science and Computer Information Systems, University of Houston Clear Lake, Houston, Texas 77058, USA; 5 Texas A&M Transportation Institute, Texas A&M University, College Station, Texas 77843, USA; 6 Industrial and Systems Engineering, Texas A&M University, College Station, Texas 77843, USA

**Keywords:** Human behaviour, Cognitive neuroscience

## Abstract

We describe a multimodal dataset acquired in a controlled experiment on a driving simulator. The set includes data for *n*=68 volunteers that drove the same highway under four different conditions: No distraction, cognitive distraction, emotional distraction, and sensorimotor distraction. The experiment closed with a special driving session, where all subjects experienced a startle stimulus in the form of unintended acceleration—half of them under a mixed distraction, and the other half in the absence of a distraction. During the experimental drives key response variables and several explanatory variables were continuously recorded. The response variables included speed, acceleration, brake force, steering, and lane position signals, while the explanatory variables included perinasal electrodermal activity (EDA), palm EDA, heart rate, breathing rate, and facial expression signals; biographical and psychometric covariates as well as eye tracking data were also obtained. This dataset enables research into driving behaviors under neatly abstracted distracting stressors, which account for many car crashes. The set can also be used in physiological channel benchmarking and multispectral face recognition.

## Background & Summary

Cars are iconic products of modern civilization, providing mobility that has transformed business and lifestyles. As a result, car driving has been engrained into people’s routines. It is an empowering activity, but also a dangerous one, as attested by the thousands of traffic crashes that take place every day.

From surveys reporting the causes of traffic crashes^1,2^, one could identify three major factors: cognitive, emotional, and sensorimotor stressors that overtax the driver’s physiological resources, thus opening the car-driver feedback loop. In fact, it has been documented that even when these stressors do not result in a crash, they degrade traffic efficiency^3^.

Among the three possible stressor types, sensorimotor stressors have been studied more extensively, because their presence and effects can be readily identified^4^. Texting, the most well-known sensorimotor stressor, has become public enemy number one. Because in texting the driver takes away her/his eyes from driving to interact with her/his cell phone, eye trackers proved to be useful sensing elements in gauging driving behaviors^5^. Eye tracking, however, is inadequate for more esoteric stressors, such as cognitive and emotional ones, where the driver may appear to maintain attention to the road, but her/his mind is intensely somewhere else. To address this issue, investigators resorted to the use of electroencephalography (EEG)^6^—a powerful, but somewhat obtrusive sensing modality.

Another crash causing factor that has gained attention in recent years, is startle. Startling events are often the result of car malfunction, such as the case of unintended acceleration. It has been postulated that the ability of the driver to handle such events is compromised by the presence of stressors.

Here, we describe two experiments, known as SIMULATOR STUDY 1, designed to provide some answers regarding stress and startle related crashes. The experiments were conducted in a driving simulator using the same subject pool:

**EXPERIMENT I** focused on the effect of cognitive/emotional/sensorimotor stressors on driving behaviors under typical conditions.

**EXPERIMENT II** focused on the effect of stress on reactivity to a startling event while driving; this startling event was unintended acceleration.

EXPERIMENT I followed a crossover design where all subjects underwent all treatments, including a control treatment (i.e., stress-free driving), on the same segment of highway and in similar traffic/weather conditions. The treatment order was randomized to ameliorate the practice effect. This design provides the opportunity to account for intra-individual differences.

EXPERIMENT II followed a parallel group design, with nearly half of the subjects assigned to the Nonloaded Group, while the other half to the Loaded Group; both groups followed the same itinerary under similar traffic/weather conditions. The Nonloaded Group had a stressor-free drive throughout the itinerary, towards the end of which they experienced the startling event. The Loaded Group had a stressor-free drive only in the first portion of the itinerary; in the second portion of the itinerary, the Loaded Group subjects were experiencing a strong stressor of mixed nature. As in the case of the Nonloaded Group, the startling event took place towards the end of the itinerary and while this time the mixed stressor was in effect. A crossover design would be inadvisable for EXPERIMENT II, as multiple applications of the startle would have incurred a level of habituation.

To the best of our knowledge, this is the first study that tackles all three types of distracting stressors under typical conditions as well as their interaction with startling events. Moreover, a unique aspect of the study is its comprehensive measurement set. This measurement set includes multiple physiological and observational variables that track sympathetic state, multiple indicators of driving performance, and a host of biographic and psychometric covariates. All of the physiological and observational variables have been extracted unobtrusively, using either thermal and visual cameras or wearable sensors. This ensured that the drivers exhibited natural behaviors, undisturbed by the mechanics of the experiments.

The presented dataset provides a rich resource for the scientific community to perform exploratory and hypothesis-driven investigations regarding the effect of various types of stressors on the internal and apparent state of drivers, as well as their driving performance. Analyzing a subset of this data, we reported in ref. 7 the existence of an autonomic mechanism that successfully counter-balances erroneous motor reactions in the presence of cognitive and emotional stressors, but breaks down when sensorimotor stressors are introduced. That work gives a small flavor of the dataset’s potential for consequential behavioral research.

Due to physiological channel redundancy, the dataset also offers the opportunity for realistic comparative studies of affective measurement methods, such as perinasal EDA versus palm EDA^8^, or benchmarking heart rate extracted from visual facial video^9^ versus heart rate extracted from thermal facial video^10^. Last but not least, thanks to the use of both thermal and visual imaging modalities for the subjects’ faces, the dataset can be used in multispectral face recognition research^11^. In this respect, a unique feature of the dataset is the abundance of facial expressions and thermophysiological changes due to bouts of stress.

## Methods

### Ethics statement

The experimental procedures were approved by the Institutional Review Boards (IRB) of the University of Houston (protocol #15028-01) and the Texas A&M University (study #IRB2014-0532). The authors performed these procedures in accordance with the approved guidelines, obtaining informed consent from each subject before conducting the experiments. In the consent form, the subjects were given two explicit data release options, for which a separate signature was required:

#### Option A

Subjects that selected this option consented to the research use of all their experimental data, but did not consent to the public release of identifying experimental data. Hence, this excluded from the public version of the dataset all their video data (i.e., facial visual video, facial thermal video, perinasal region of interest video, and operational theater video).

#### Option B

Subjects that selected this option consented both to the research use and public release of all their experimental data, including video data bearing identifying information. Only two subjects selected OPTION A (Subject T046 & Subject T074). All other subjects selected OPTION B.

### Subjects

We recruited subjects from the Bryan and College Station, TX communities (population about 250,000) through email solicitations and flyer postings. Subjects had a valid driving license and had normal or corrected to normal vision. We restricted admission to individuals with at least one and a half years of driving experience who were between 18 and 27 years of age (Young cohort) or above 60 years of age (Old cohort). We excluded subjects on medications affecting their ability to drive safely. A total of *n*=78 subjects conforming to the inclusion-exclusion criteria volunteered for the study. One subject quit the experiment because of motion sickness; and, raw data for *n*=9 subjects were not recorded properly due to technical issues. Hence, raw data for *n*=68 subjects (35 male/33 female) were nearly complete, comprising our working set. For this working set, we could not reliably extract perinasal perspiration signals from *n*=9 male subjects, because they had facial hair. All other variables in the working set were acquired without significant problems.

### Experimental setup

**Figure 1:**
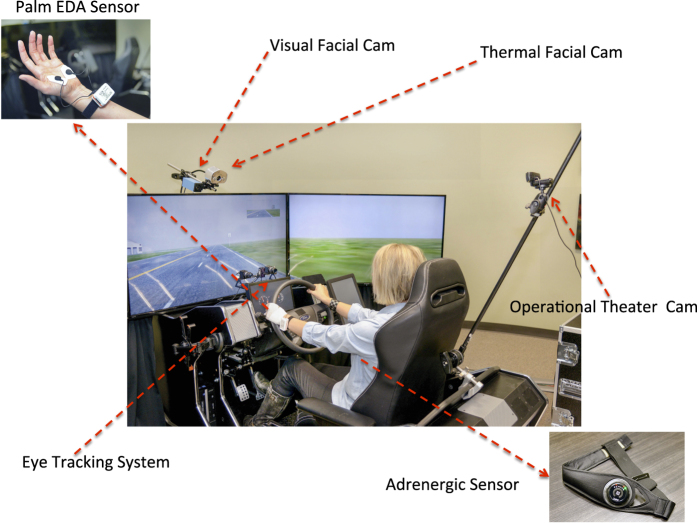
Experimental setup.

We conducted the experiment in a driving simulator manufactured by Realtime Technologies, Inc, Royal Oak, MI. During the experimental sessions, we continuously imaged the subject’s face with a thermal camera as well as a visual camera (Fig. 1). Specifically:

#### Thermal facial camera

We used a Tau 640 long-wave infrared (LWIR) camera (FLIR Commercial Systems, Goleta, CA); it features a small size (44×44×30 mm) and adequate thermal (<50 mK) and spatial resolution (640×512 pixels). The Tau 640 camera was outfitted with a LWIR 35 mm lens f/1.2. Thermal data was collected at a frame rate of 7.5 fps. We used these thermal facial videos to extract perinasal perspiratory signals, known to commensurate with electrodermal (EDA) activity in the palm^8^. For this reason we call the thermally extracted perinasal perspiration signals, *perinasal EDA signals*.

#### Visual facial camera

We used a FireWire CCD monochrome zoom camera (The Imaging Source, Charlotte, NC) with spatial resolution 1,024×768 pixels. Visual data was collected at a frame rate of 15 fps. We used these facial visual videos to extract emotional signals, based on the analysis of facial expressions. The visual camera was placed next to the thermal camera to facilitate spatial co-registration (Fig. 1).

The facial cameras were at a distance of approximately 1.2 m from the subject. This distance in combination with the camera optics ensured that a typical face covered a significant portion of each thermal and visual frame, providing maximum spatial resolution for image analysis (see Supplementary Fig. 1b in ref. 7).

A second visual camera, termed *operational theater camera*, was placed behind the driver, aiming at the simulator’s central screen, in order to record the subject’s unfolding drive (Fig. 1). This was an HD Pro Webcam C920 (Logitech, Newark, CA) with spatial resolution 1,920×1,080 pixels, collecting data at a frame rate of 30 fps.

We collected additional physiological data via two wearable sensors. Specifically:

#### Palm EDA sensor

We used the Shimmer3 GSR sensor (Shimmer, Dublin, Ireland) to collect palm EDA data (Fig. 1). The sensor is powered with a rechargeable Li-ion battery and its measurement range is 10–4,700 kΩ; it wirelessly transmits data to the host computer via a Bluetooth connection. The palm EDA signal collected via the Shimmer device and the perinasal perspiration signal extracted from the thermal facial imagery, are indicators of cholinergic control.

#### Adrenergic sensor

We used the Zephyr BioHarness 3.0 (Zephyr Technology, Annapolis, MD) sensor to measure the subject’s heart rate and breathing rate—two standard indicators of adrenergic control. The sensor connects to a chest strap that is worn underneath the subject’s clothing (Fig. 1). It is powered by a rechargeable lithium polymer battery (up to 26 h per charge), and is capable of detecting a heart rate range of 25–240 bpm and a breathing rate range of 4–70 bpm.

We collected eye tracking data with faceLAB (Seeing Machines, Canberra, Australia). The faceLAB eye tracking system uses dashboard-mounted cameras (Fig. 1), and a small pod illuminating the subject’s face with infrared light. The amount of light used is well within the safe limits of exposure and is almost indiscernible. The faceLAB’s software displays the subject’s point of gaze vectors; the footprint of these gaze vectors on the central simulator screen is a moving green dot.

Importantly, we programmed the simulator to save a record of the evolving driving parameters. These parameters included speed, acceleration, brake force, steering angle, and lane position.

### Experimental design

Following a randomized block design, we assigned subjects to two groups: Nonloaded Group (*y*=*o*, *n*=35) and Loaded Group (*y*=*L*, *n*=33). This group categorization related to the two arms of EXPERIMENT II. All sessions in EXPERIMENT I were the same for both groups. Upon signing the consent form, the subjects completed three questionnaires:

#### Biographic questionnaire

It identified key facts about the subject, such as gender, age, and driving record.

#### (State-) Trait anxiety inventory

Long-standing stress might have an effect on sympathetic responses and thus, scoring trait anxiety is of potential interest to research relevant to this dataset^12^.

#### Personality type A/B

This was a modified version of the Jenkins Activity Survey^13^. Some studies have shown association between type A personalities and specific driving behaviors^14^; thus, scoring of type A/B personalities is also of potential interest to research relevant to this dataset.

Next, the subjects went through *T*_*session*_=8 experimental sessions. The first seven sessions constituted EXPERIMENT I, and were crossovered. The eighth session had two arms and the subjects were parallel grouped (EXPERIMENT II).

### Experiment I

The first session of EXPERIMENT I was a baseline session (1: B) where the subjects sat quietly in a dimly lit room, listening to soothing music for 5 min. Following this baseline session, the subjects went through six driving sessions on the simulator. In order of execution, the drives were as follows:

#### 2: Practice drive (PD)

The subjects familiarized themselves with the driving simulator by driving on a 8 km straight section of a four-lane highway at posted speeds; two lanes were dedicated to traffic in each direction, with the subject’s car traveling in the right lane (R); the speed limits changed every couple of kilometers (80 km/h→50 km/h→100 km/h)—see Supplementary Fig. 2 in ref. 7.

#### 3: Relaxing drive (RD)

The subjects had to drive on a 10.9 km straight section of a four-lane highway with posted speed limit of 70 km h^−1^; two lanes were dedicated to traffic in each direction, with the subject’s car traveling in the right lane (R); there was light traffic on the oncoming lanes (~3 vehicles per km). The subjects were forced to change lane (R to L) after 5.2 km into the drive. They had to stay in the left lane (L) for 1.2 km, before they had the opportunity to drive back to the right lane (R), if they wished—see Supplementary Fig. 3 in ref. 7. The rationale for this lane change was to reduce the monotony of the drive.

#### 4–7: Loaded drives

We randomized the order of four special driving sessions, called ‘loaded’ drives, featuring the same challenging driving conditions (construction zones—see Supplementary Fig. S4 in ref. 7). Each loaded drive was uniquely characterized by an additional stressor or absence thereof. This stressor assumed the form of a secondary activity that was forced in two phases during the course of the drive. All loaded drives were on the same 10.9 km section of a four-lane highway with posted speed limit of 70 kph; two lanes were dedicated to traffic in each direction, with the subject’s car traveling in the right lane (R). The drives featured heavy traffic on the oncoming lanes (>12 vehicles per km), construction on the left lane (L), and traffic delineator posts on both sides of the right lane (R). The subjects were forced to change lane (R to L) after 4.4 km into the drive. They stayed in the left lane (L) for 1.2 km, before they were directed back to the right lane (R). During the detour, construction cones appeared on the right side of the lane. In more detail, the loaded drives were as follows:

- **Loaded Drive:** (LD_*Ø*_ ≡ ND) Driving with no secondary activity (no additional stressor); it is also known as Normal drive.
- **Cognitive Drive:** (LD_*C*_ ≡ CD) Driving under a cognitive stressor. The cognitive stressor was mathematical questions (see in ref. 7, Supplement: Cognitive Stressor—Mathematical Questions) in one phase of the drive and analytical questions (see in ref. 7, Supplement: Cognitive Stressor—Analytical Questions) in another phase of the drive, posed orally by the experimenter. The experimenter started posing these questions from the beginning of the relevant list, stopping only when the phase was over. The subjects had to answer the questions to the best of their abilities. The mathematical versus analytical phase order was randomized.
- **Emotional Drive:** (LD_*E*_ ≡ ED) Driving under an emotional stressor. The emotional stressor was emotionally stirring questions posed orally by the experimenter in two phases. There were two sets of questions: a set with less pointed questions (see in ref. 7, Supplement: Emotional Stressor—Basic Questions) and a set with more pointed questions (see in ref. 7, Supplement: Emotional Stressor—Pointed Questions). In the first stressful phase, the experimenter was asking basic questions for 20 s, starting from the beginning of the relevant list. In the remaining time of the first stressful phase, the experimenter was asking pointed questions, starting from the beginning of the corresponding list. In the second stressful phase, the experimenter continued for 30 s with basic questions, starting form the point s/he left in the first stressful phase. In the remaining time of the second stressful phase, the experimenter was asking pointed questions, starting from the point s/he left in the first stressful phase. The subjects had to answer all these questions to the best of their abilities.
- **Sensorimotor Drive:** (LD_*M*_ ≡ MD) Driving under a sensorimotor stressor. The sensorimotor stressor was texting back words, sent one by one to the subject’s smartphone; this texting exchange took place in two phases.

The phase layout within each stressful LD_*j*_ drive (*j*∈[*C*,*E*,*M*]) was as follows:

- **Phase** $P1_{LD_{j}}$: Driving without distractions for ~80 s.
- **Phase** $P2_{LD_{j}}$: Driving while engaging in a secondary activity *j* for ~160 s.
- **Phase** $P3_{LD_{j}}$: Driving without distractions for ~240 s (coincided with the detour).
- **Phase** $P4_{LD_{j}}$: Driving while engaging in a secondary activity *j* for ~160 s.
- **Phase** $P5_{LD_{j}}$: Driving without distractions for ~120 s.

### Experiment II

#### 8: Failure drive (FD)

Subjects had to drive a 3.2 km highway section identical to the last 3.2 km segment of the loaded drives. Subjects belonging to the *y*=*o* group did not engage in any secondary activity (see Supplementary Fig. 5 in ref. 7). Subjects belonging to the *y*=*L* group, however, drove under mixed stressors the last 2 km of the drive (see Supplementary Fig. 6 in ref. 7). Initially, *y*=*L* subjects had to text back a sentence that appeared in their smartphone; then, they had to respond to an alternating series of mathematical/analytical and emotional questions posed orally by the experimenter while they kept texting. Towards the end of the drive, all subjects had to wait on a red light at an intersection. Prior to the green signal a vehicle malfunction resulted into an unintended acceleration incident, propelling the car forward and putting it on a collision course with another car that had entered the intersection. The subject had 5 s to react before a collision. Hence, the FD drive had three phases ${\mathrm{Pi}}_{FD_{y}}$ (*i*∈[1, 2, 3], *y*∈[*o*, *L*]):

- **Phase** $P1_{FD_{y}}$: First portion of drive—no distractions.
- **Phase** $P2_{FD_{y}}$: Second portion of drive—*y*=*o* no distractions; *y*=*L* mixed distractions.
- **Phase** $P3_{FD_{y}}$: Experiencing an unintended acceleration incident for ~11 s.

There was a 2 min break between drives. Starting with the Relaxing drive (RD), during each break subjects were completing the NASA Task Load Index (TLX) for the preceding drive. NASA-TLX is a subjective workload assessment tool that complements the objective assessment of task-induced sympathetic arousal, captured via physiological sensing. NASA-TLX features a multi-dimensional rating procedure that derives an overall workload score based on a weighted average of ratings on six sub-scales. These sub-scales include Mental Demand, Physical Demand, Temporal Demand, Own Performance, Effort, and Frustration^15^.

### Computation

Algorithmic processing of the thermal imagery yielded a signal that quantified perinasal perspiration. The algorithm included a virtual tissue tracker that kept track of the region of interest, despite the subject’s small motions. This ensured that the thermophysiological signal extractor operated on consistent and valid sets of data over the clip’s timeline.

### Thermal imaging—tissue tracking

We used the tissue tracker reported by Zhou *et al.*^16^. On the initial frame, the user initiates the tracking algorithm by selecting the upper orbicularis oris portion of the perinasal region. The tracker estimates the best matching block in every next frame of the thermal clip via spatio-temporal smoothing (see Supplementary Fig. 7 in ref. 7). A morphology-based algorithm is applied on the evolving region of interest to compute the perspiration signal.

### Thermal imaging—perinasal eda signal extraction

In ref. 7, Supplementary Fig. 7 shows the thermal signature of perspiration spots on the perinasal area of a subject in moments of low and high excitation. In facial thermal imagery, activated perspiration pores appear as small ‘cold’ (dark) spots, amidst substantial background clutter. The latter is the thermophysiological manifestation of the metabolic processes in the surrounding tissue. We quantified this spatial frequency pattern by extracting an energy signal **E**(*k*, *j*, *i*), indicative of perspiration activity in the perinasal area of subject *k*, for session *j*, and phase *i*. We computed this signal by applying the clinically validated morphological method reported by Shastri *et al.*^8^. Any high-frequency noise in this signal was suppressed by a Fast Fourier Transformation (FFT) filter.

### Code availability

We used the S-Interface (formerly OTACS) software to extract the perinasal EDA signals. The S-Interface is a modular software system that reads radiometric (i.e., raw thermal) files, applies certain operations on them according to the software plug-ins in its current configuration, and outputs the result (ref. 17). In this case, the S-Interface configuration included:

1. The tracker plug-in (an implementation in C# of ref. 16), which was following the perinasal region of interest (ROI), nullifying the effect of head motion.
2. The perspiratory morphological signal extractor (an implementation in C# of ref. 8), which was operating upon the orthorectified perinasal ROI, yielding a signal commensurate to the extend of activated perspiration pores.

We extracted signals of basic emotions by processing the facial videos via the Computer Expression Recognition Toolbox (CERT)^18^.

## Data Records

The data is freely available on the Open Science Framework (Data Citation 1). The repository’s data is organized per subject under three major directories: (1) Raw Thermal Data—1.54 TB in size. (2) Structured Study Data—57.5 GB in size. (3) R-Friendly Study Data—40.1 MB in size. In these directories, the subject folders are named Txxx, where xxx stands for the subject number.

At the repository’s root, a spreadsheet named ‘Dataset-Table-Index.xlsx’ gives an exhaustive enumeration of the dataset’s files in the Raw Thermal Data and Structured Study Data directories. Files expected to be present and are present indeed in these two directories, are denoted by ‘1’; there are 4,905 such files. Files expected to be present, which are not present for technical and other reasons, are denoted by ‘0’; there are 234 such cases. Files that are not supposed to be present due to the experimental design are denoted by ‘NA’; there are 544 such cases. Files that have been redacted due to IRB restrictions, are denoted by ‘IRB’; there are 40 such cases. Files associated with derivative thermal variables, such as perinasal EDA, which could not be extracted due to the presence of facial hair, are denoted by ‘N’; there are 144 such cases. Files that are present, but found during the technical validation process to be marred by noise, are denoted by ‘−1’; there are 117 such files and we do not recommend using their data.

The R-Friendly Study Data directory is a reformatting of the quantitative variables contained in the Structured Study Data directory. It contains, however, one post-study curated variable that is not present in the Structured Study Data directory. This variable is the eye tracking variable, and the ‘Dataset-Table-Index.xlsx’ spreadsheet lists its present (426) and missing (118) data files in a specially highlighted column.

### (1) Raw thermal data directory

In the Raw Thermal Data directory, each subject’s folder contains the facial thermal sequences for all experimental sessions. These binary files are named ‘Txxx-SESSION_CODE.dat’, where xxx stands for the subject number and SESSION CODE holds the acronym of the experimental session, that is, B for the Baseline, PD for the Practice Drive, RD for the Relaxing Drive, ND for the Normal Drive, CD for the Cognitive Drive, ED for the Emotional Drive, MD for the Sensorimotor Drive, and FDL or FDN for the Failure Drive. Each .dat file is accompanied by an .inf file in text format. The header of each .inf file has three numbers: The first number denotes the number of thermal frames contained in the corresponding .dat file; the second number denotes the width of each thermal frame; and, the third number denotes the height of each thermal frame. The body of each .inf file contains the timestamps of all thermal frames contained in the corresponding .dat file. The .dat files can be accessed via the S-Interface^17^, which uses the information in the corresponding.inf files to properly open .dat files and process them.

### (2) Structured study data directory

In the Structured Study Data directory, each subject’s folder has three files containing biographic and psychometric data; they are colocated with subfolders containing data corresponding to experimental sessions, thus forming a hierarchical data sturcture reflective of the experimental design. Specifically:

#### Txxx.b: Biographic data—session independent

This Excel file contains Gender {Male, Female} information; Age {Integer} information; and, Age Group {Old, Young} information.

#### Txxx.bar: Psychometric data—all sessions

This Excel file contains the NASA TLX scores the subject provided after each experimental session, following the Relaxing Drive (RD). NASA TLX is a multi-dimensional psychometric^15^, measuring perceived Mental Demand, Physical Demand, Temporal Demand, Performance, Effort, and Frustration.

#### Txxx.tp: Trait psychometric data—session independent

This Excel file contains the (State-) Trait Anxiety Inventory (STAI)^12^ score and the Type A/B Personality^13^ score.

The subfolders containing data pertaining to experimental sessions (treatments), are named ‘Y SESSION_CODE’, where Y stands for the order of the session in the experimental timeline and SESSION_CODE holds the acronym of the session. The Baseline [B], Practice Drive [PD], Relaxing Drive [RD], and Failure Drive—either Loaded [FDL] or Nonloaded [FDN], have constant order across subjects: B→1, PD→2, RD→3, FDL or FDN→8. The loaded drives, that is, Normal Drive [ND], Cognitive Drive [CD], Emotional Drive [ED], and Sensorimotor Drive [MD] have randomized order, blocked by gender and age group; thus, their order, drawing from the set [4…7], differs from subject to subject.

Each session folder contains up to nine files holding measurements from different modalities, and a stimulus file, where needed, holding information about the stressors/events that applied/took place during the session. Specifically, the stimulus file is named ‘Txxx-zzz.stm’, where xxx is the subject number and zzz is the session’s order number. Each stressor/event is specified in a separate row that lists its: Start Time [in s], End Time [in s], and Type [Other ≡ 0, Analytical Questions ≡ 1, Mathematical Questions ≡ 2, Emotional Questions ≡ 3, Texting ≡ 4, Texting and Talking ≡ 5, Failure Event ≡ 6].

The multimodal measurements for each experimental session are held in files named Txxx-zzz.MEASUREMENT_CODE, where xxx is the subject number, zzz is the session’s order number, and MEASUREMENT_CODE refers to the measurement channel. Specifically:

#### Txxx-zzz.BR: Breathing rate signal

This Excel file contains three synced columns: Frame #; Time; and, Breathing Rate signal. Hence, scanning each row from left to right we find the chronological rank order of the instantaneous measurement, the time [in s] the measurement was taken with respect to the beginning of the session, and the value of the breathing rate signal at that time [in bpm]. No breathing rate measurements were recorded during the baseline (B) sessions.

#### Txxx-zzz.HR: Heart rate signal

This Excel file contains three synced columns: Frame #; Time; and Heart Rate signal. Hence, scanning each row from left to right we find the chronological rank order of the instantaneous measurement, the time [in s] the measurement was taken with respect to the beginning of the session, and the value of the heart rate signal at that time [in bpm]. No heart rate measurements were recorded during the Baseline (B) sessions.

#### Txxx-zzz.peda: Palm EDA signal

This Excel file contains three synced columns: Frame #; Time; and, Palm EDA signal. Hence, scanning each row from left to right we find the chronological rank order of the instantaneous measurement, the time [in s] the measurement was taken with respect to the beginning of the session, and the value of the palm EDA signal at that time [in kΩ]. No palm EDA measurements were recorded during the Baseline (B) sessions.

#### Txxx-zzz.pp: Perinasal EDA signal

This Excel file contains four synced columns: Frame #; Time; Perinasal EDA signal; and, Noise Reduction (NR) Perinasal EDA signal. Hence, scanning each row from left to right we find the chronological rank order of the instantaneous measurement, the time [in s] the measurement was extracted with respect to the beginning of the session, the value of the perinasal EDA signal at that time [in °C^2^], and its smoothed over value [also in °C^2^]. Perinasal EDA measurements were the only physiological measurements recorded during the Baseline (B) sessions.

#### Txxx-zzz.avi2: Perinasal region of interest thermal video

This avi file is the thermal video recording of the perinasal region of interest (ROI) during session zzz. The S-Interface extracts this ROI video out of the raw thermal facial imagery, thanks to the tracker reported in ref. 16. It is upon this tracked ROI that the physiological signal extractor reported in ref. 8 operates, yielding the perinasal EDA signal.

#### Txxx-zzz.res: Performance response variables

This Excel file contains 7 synced columns: Frame #; Time; Speed signal; Acceleration signal; Brake Force signal; Steering signal; and, Lane Position signal. Hence, scanning each row from left to right we find the chronological rank order of the instantaneous measurement, the time [in s] the composite measurement was taken with respect to the beginning of the session; and, the values at that time of speed [in km h^−1^], acceleration [in °], brake force [in N], steering [in rad], and lane position [in m].

#### Txxx-zzz.avi3: Operational theater video

This avi file is the first-person video recording of what the driver sees during the session zzz. The green dot identifies the direction of the driver’s gaze, superimposed on the video by the eye tracker.

#### Txxx-zzz.FACS: FACS signals

This Excel file contains 10 synced columns: Frame #; Time; Anger signal; Contempt signal; Disgust signal; Fear signal; Joy signal; Sad signal; Surprise signal; and, Neutral signal. Hence, scanning each row from left to right we find the chronological rank order of the instantaneous measurement, the time [in s] the composite measurement was taken with respect to the beginning of the session; and, the normalized values of the composite FACS vector at that time, extracted via the CERT software. These normalized values indicate levels of anger, contempt, disgust, fear, joy, sad, surprise, and neutral feelings, respectively.

#### Txxx-zzz.avi1: Facial video

This avi file is the facial video recording during session zzz. It is upon this video that the CERT software operates to extract the FACS signals.

### (3) R-friendly study data directory

The R-Friendly Study Data directory has a copy of the physiological and performance signals in a flat format, and features some additional data, too. In the flat format, all signals for a subject are arranged column-wise in a single file (no session subdirectories). Each such subject file is enhanced by the addition of columns bearing traveled distance [in m] and explicit eye tracking data (gaze x position, gaze y position, left pupil diameter, right pupil diameter). Distance and explicit eye tracking data do not exist in the original hierarchical data structure saved in the Structured Study Data directory; their curation was an afterthought. The R-Friendly Study Data directory allows easy addition of such quantitative variables to the dataset during post-study work.

## Technical Validation

Our validation method comprised three steps: (1) Quality control of variables. (2) Quality control of support media. (3) Experimental validation.

### Quality control of variables

We performed quality control regarding the expected range and patterns for each of the variables involved in the study. We will describe the quality control process and results per variable category: (a) Biographic variables. (b) Psychometric variables. (c) Physiological variables. (d) Performance variables. (e) Eye tracking variables. 2,3,4,5, 6 in this section are comprehensive, serving as quality control devices rather than instruments of detail. We provide a typical detailed example from the dataset in Supplementary Fig. 2, depicting some key variables for the Sensorimotor Drive (MD) of Subject T029.

The biographic and psychometric variables were collected in snapshots, in nominal and ordinal form, respectively. The physiological and performance variables were collected continuously throughout the experimental sessions in the form of signals. For the physiological variables, we extracted the relevant signals as time functions. For the performance variables, we extracted the relevant signals as functions of both time and distance travelled. The reason is that there were speed differences among subjects, and anchoring the performance signals to space provided a more consistent basis of comparison. The eye tracking variables were collected in the form of scatterplots (x-coordinate, y-coordinate).

#### Biographic variables

The age ranges in the working set of 68 subjects were [18, 27] and [60, 86], which were conforming to the inclusion ranges of [18, 27] and > 60. The working set was balanced with respect to the blocking variables of gender (35 male/33 female) and age group (34 Old/34 Young), as well as the two load conditions in the Failure Drive (35 Nonloaded/33 Loaded).

#### Psychometric variables

The score range in the (State-) Trait Anxiety Inventory (STAI) was [20, 52], which is within the acceptable range of [20, 80] for this instrument. The score range in the Personality Type A/B Inventory was [144, 282], which is within the acceptable range of [35, 380] for this instrument. The (State-) Trait Anxiety and Personality Type A/B Inventories are important covariates. The recorded score ranges for both STAI and A/B did not include extreme values, which suggests that the set of volunteers were normal subjects, rendering unlikely any bias in experimental respones. Indeed, in ref. 7 we reported no significant correlation of STAI and A/B scores with mean perinasal EDA responses or mean absolute steering or maximum right-side/left-side lane departures in any of the drives (*P*> 0.05 for the correlation coefficients in all cases). This suggests that key personality traits that could have biased sympathetic responses, driver reactions, and driver performance did not play any role, at least for the *n*=59 subjects for whom we had perinasal EDA signals.

For the sub-scales of the NASA-TLX index, the score ranges were as follows: Mental Demand: [1, 20]; Physical Demand: [1, 20]; Temporal Demand: [1, 20]; Performance: [1, 20]; Effort: [1, 20]; Frustration: [1, 20]. Hence, all the score ranges were within the acceptable range of [1, 20] for the sub-scales of this instrument.

#### Physiological variables

These variables include breathing rate, heart rate, palm EDA, and perinasal EDA signals, aiming to track evolving stress states. They are the main explanatory variables, as the study assumes that excessive stress induced by distractions likely affects aspects of the drivers’ performance.

**Figure 2:**
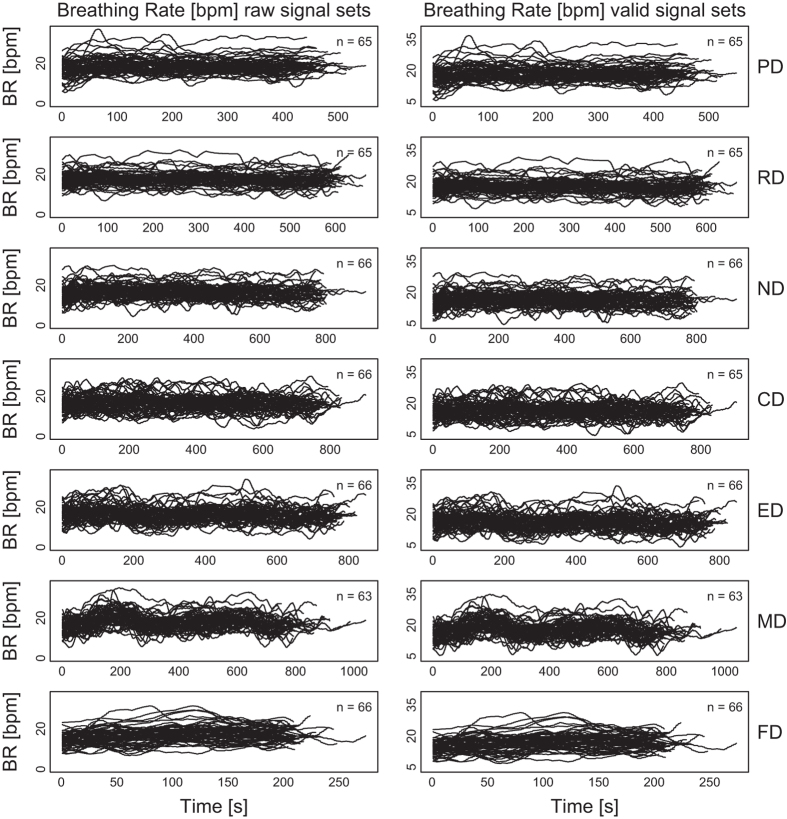
Breathing rate signals per drive, for all subjects, before and after quality control—raw and valid signal sets, respectively.

**Breathing Rate Signals:** Figure 2 shows the breathing rate signals per drive, for all subjects, before and after quality control. The quality control process involved the removal of signals featuring at least one value outside the range [4, 40] bpm. Values outside this range are possible but not likely for sitting subjects that are occasionally under moderate stress. We found 0.003% of the breathing rate data to be below 4 bpm, suggesting very loose sensor fitting. We did not find any data that exceeded 40 bpm. Breathing rate measurements with a chest strap sensor, such as the Zephyr BioHarness 3.0, are more resilient to noise with respect to heart rate measurements by the same sensor, due to the sensing mechanics for breathing; the breathing signal formation tracks the expansion and contraction of the chest’s circumference, thus ameliorating the effect of relatively poor fitting and posturing.

**Figure 3:**
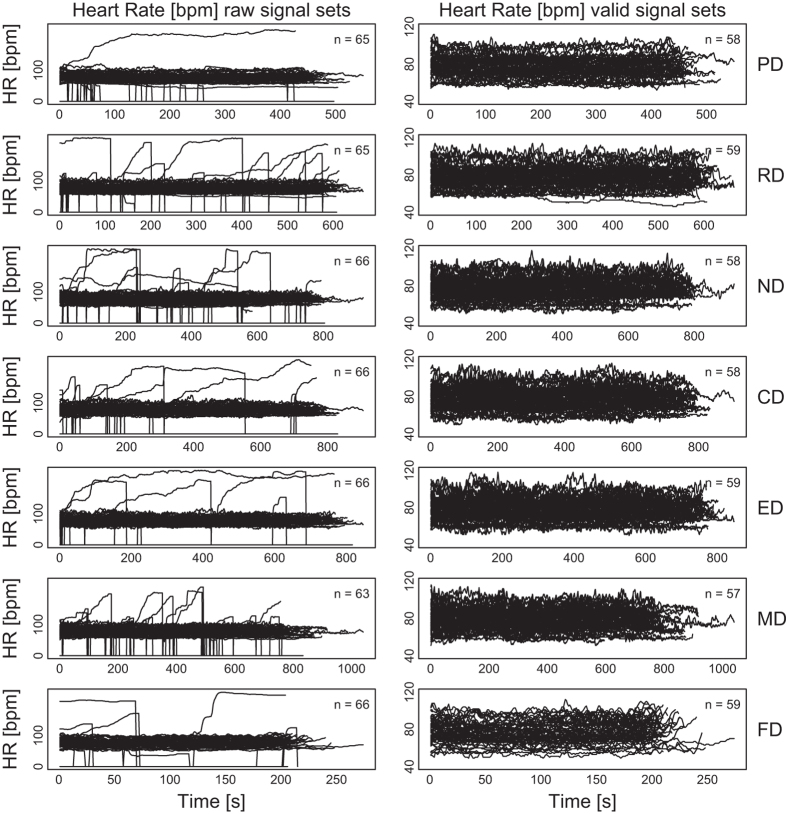
Heart rate signals per drive, for all subjects, before and after quality control—raw and valid signal sets, respectively.

**Heart Rate Signals:** Figure 3 shows the heart rate signals per drive, for all subjects, before and after quality control. The quality control process involved the removal of signals featuring at least one value outside the range [40, 120] bpm. Values outside this range are possible but not likely for sitting subjects that are occasionally under moderate stress. Importantly, signals with values outside the [40, 120] bpm range tended to drift downwards or upwards, depending on the range limit that were exceeding, respectively. Drifting downwards to very low values is an indication that the chest strap sensor has been loosing contact with the subject’s skin due to posturing, while drifting upwards to very high values is an indication that the chest strap sensor has been sliding against the subject’s skin due to poor fitting. We found 5.2% of the heart rate data to be below 40 bpm, while 2.3% of the data to be above 120 bpm; all of drifting nature. Hence, these problems were of small scale and consistent with wearability limitations for chest strap sensors reported in the literature.

**Figure 4:**
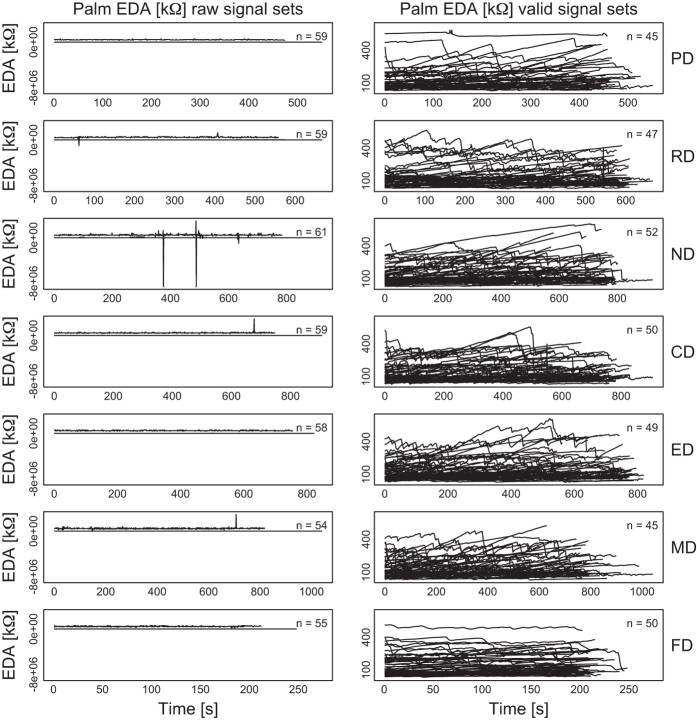
Palm EDA signals per drive, for all subjects, before and after quality control—raw and valid signal sets, respectively.

**Palm EDA Signals:** Figure 4 shows the palm EDA signals per drive, for all subjects, before and after quality control. The quality control process involved the removal of signals featuring at least one value outside the range [28, 628] kΩ. Values outside this range are possible but not likely, suggesting poor sensor fitting. We found 0.01% of the palm EDA data to be below 28 kΩ, while 8.7% of the data to be above 628 kΩ. Taken into account that the subjects’ hands were handling the steering wheel, the percentage of non-valid data was consistent with the wearability constraints for this type of sensor.

**Figure 5:**
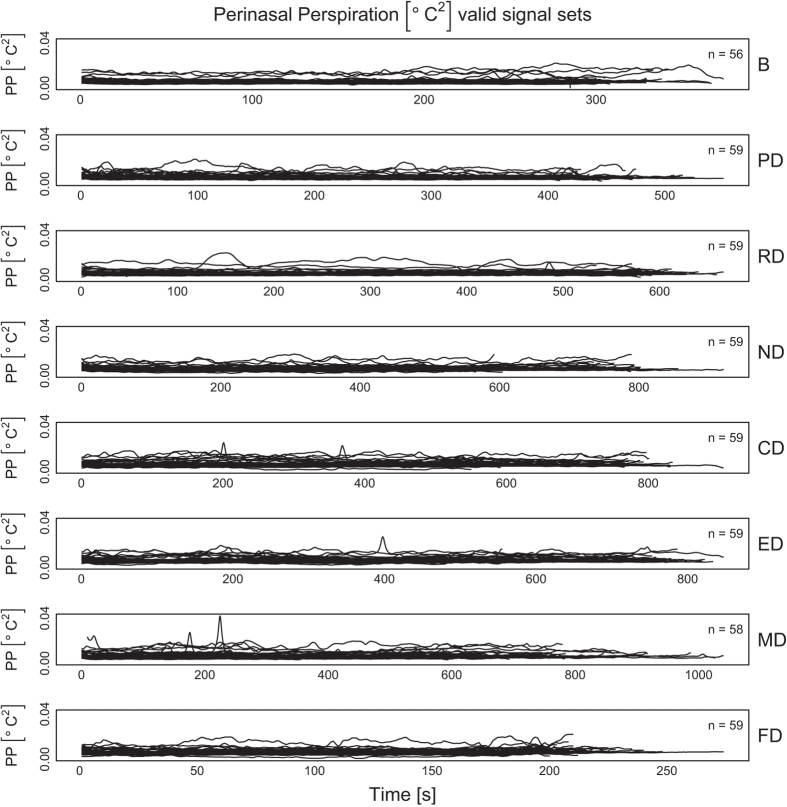
Perinasal NR EDA signals per drive, for all subjects.

**Perinasal EDA Signals:** Figure 5 shows the perinasal NR EDA signals per session, for all subjects. This is the only physiological variable for which measurements were recorded not only in the experimental drives, but also in the baseline (B) session. No further screening was necessary for the perinasal EDA channel, as quality control in this case is assured by the algorithmic signal extraction process (see Methods section). In fact, NR stands for Noise Reduction signal, and is the outcome of an FFT filter on the original perinasal EDA signal^8^.

### Performance variables

**Figure 6:**
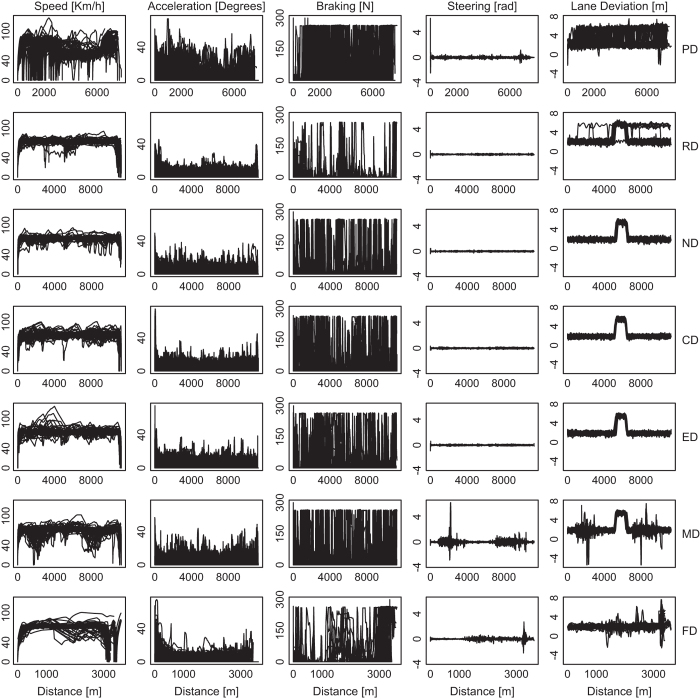
Performance signals per variable and drive, for all subjects.

These variables include the car’s speed, acceleration, brake force, steering, and lane position signals. They are the response variables, as the key questions that motivated this study center around issues of driver performance under distracting stressors. The matrix of panel graphs in Fig. 6 show the performance signals for all subjects per variable and drive. These visualizations convey valuable quality control information.

**Speed Signals:** Due to the sensitivity of the simulator around zero speed, we replaced all values between −0.1 and 0.1 [kph] in the speed signals with zero (1.3% of all speed values). Speed values lower than −0.1 [kph], we treated them as missing (0.03% of all speed values).

In the Relaxing Drive (RD) and in most Loaded drives (ND, CD, ED), we observe that between the start and end points the speed signals remain largely around the posted speed limits. This makes sense, as these drives were on a straight highway with free-flowing traffic. The Sensorimotor Drive (MD) tends to depart from this pattern, due to the strong effect of the physical distraction, which is clearly visible in two phases. Moreover, we observe that for all drives the speed signals start from 0 km h^−1^, as the car was at full stop in the beginning. The picture is more nuanced at the end of the drives. Ideally, the Relaxing (RD) and Loaded drives (ND, CD, ED, MD) were meant to end at a red traffic light, 10.9 km away from the start location. The end was signified by the appearance of a stop sign in the middle of the simulator screen, after which all recording automatically stopped. Due to small differences in the exact starting point from drive to drive and from subject to subject, in some drives the itinerary ended mostly just before the traffic light, while in some other drives the itinerary ended mostly just after the traffic light. In ND and ED, where the itinerary ended mostly before the traffic light, many subjects were caught driving at a cruising speed. Only a few subjects caught at the red light, and we can observe a thin vertical drop down to zero for the speed signal endings in ND and ED, due to this. In RD, CD, and MD, where the itinerary ended mostly after the traffic light, many subjects were caught as they nearly reached cruising speed again, at various distances from the traffic light. These partially overlapped ‘V’ shape endings created a more spread out vertical ‘wall’ in the speed signal panels of RD, CD, and MD.

In the Practice Drive (PD), the speed is not uniformly maintained around the posted speed limit and this is to be expected. The purpose of PD was to familiarize the subjects with the driving simulator, and they were explicitly instructed to test acceleration, brake force, and maneuverability at will. In the Failure Drive (FD), due to the unintended acceleration event at the end, the speed goes from 0 km h^−1^ back up to a high value, and this pattern occasionally repeats once, as some drivers end up in the ditch and try to get out of there. There is also notable undulation in the second half of FD, due to the strong effect of the mixed stressor in the loaded cohort.

**Acceleration Signals:** In the simulator, the acceleration pedal is connected to a simulated throttle valve that can move from a full close (0°) to a full open (90°) position. Due to the sensitivity of the simulator’s acceleration pedal, when the driver releases it, the system may occasionally record negative acceleration values, which are meaningless and have to be removed. In the curation process, we found that 0.3% of the acceleration signal data had negative values, and we replaced them with missing values.

We observe that for all drives the acceleration signals are very intense in the beginning, as the cars speed up to reach the posted speed limit. At the end of the drives, things differ a little bit depending on the mix of instances. In some instances the itinerary ended just before the traffic light, while the subjects were driving at a cruising speed and the acceleration signals were subdued. In some other instances the itinerary ended just after the traffic light, while the subjects were speeding up, after waiting on a green light, and thus the acceleration signals were nearly as intense as in the start of the drive.

In the Failure drive (FD), we observe zero acceleration extended for a short period towards the end. This is when the unintended acceleration event took place. The strong acceleration associated with that event was the result of a simulated engine malfunction, not a pedal action. Consequently, it was not captured by the acceleration sensor, which monitors the driver’s pedal only. The end effect of this unintended acceleration, however, was captured by the speed sensor and manifests in the corresponding speed signals as rapid speed increase. In the Practice Drive (PD), the acceleration signals are intense throughout the drive, as drivers were braking and accelerating multiple times at will, testing the system.

**Brake Force Signals:** The maximum braking force is 300 [N]. In 0.004% of the signal data, however, we found the braking force value to exceed 300 [N]. In these cases the driver was in the start position, pressing the brake, and had simultaneously the hand brake on. To simplify things, we replaced all braking force values found to be higher than 300 [N] with 300 [N].

We observe that for the Relaxing Drive (RD), braking is relatively sparse. This is to be expected for a straight itinerary with no road construction and no stressors, rendering itself to a ‘cruise control’ type of driving. In the Loaded drives (ND, CD, ED, MD) braking is more frequent due to the road construction and stressor effects. The same is true for the Practice Drive (PD), but for a different reason—subjects were honing their simulator driving skills.

**Steering Signals:** We observe that the steering signals feature small and symmetric fluctuations around zero for the Relaxing Drive (RD), Normal Drive (ND), Cognitive Drive (CD), and Emotional Drive (ED). This is to be expected for mostly straight itineraries, where the drivers are not physically distracted. In the Practice Drive (PD) there is relatively bigger steering undulation, due to deliberate freewheeling. For example, a notable outlier in the beginning of PD is due to subject T073 steering sharply off the road. In the Sensorimotor Drive (MD), where there is physical distraction before and after the detour, we observe that the intensity of the steering signals gets bigger and occasionally asymmetric in those phases^7^. The same is true for the second portion of the Failure Drive (FD), where about half of the drivers (loaded cohort) were distracted with a mixed stressor. It is also true for almost all steering signals at the end of FD, as the drivers were trying to avoid collision due to unintended acceleration.

**Lane Position Signals:** The lane position signals track the position of the center of the car from the right edge of the rightmost lane, which is the driving lane used by the subjects in this study. The width of each lane is 3.65 m and the car’s width is 1.85 m. Car positions to the left of the reference edge assume positive values, while car positions to the right of the edge, assume negative values. Obviously, negative values indicate dangerous lane deviations (off the paved road), while large positive values (>(3.65–1.85/2)=2.725 m) indicate incursions to neighboring lanes.

We observe orderly, middle of the lane trajectories in the Normal (ND), Cognitive (CD), and Emotional (ED) drives, mirroring the shape of the highway with its characteristic detour in the middle of the itinerary. This orderly picture disintegrates in the Sensorimotor Drive (MD), where there are significant lane departures due to non-balanced handling of the steering wheel at times of high undulation—a direct result of physical distractions^7^. The same is true for the second half of the Failure Drive (FD). In the Practice Drive (PD) there is an almost uniformly scattered picture, as the subjects were driving in a freewheeling manner. In the Relaxing Drive (RD) several drivers did not return back to the rightmost lane after the detour but continued driving on the leftmost lane; some drivers changed lanes even before the detour. This is because there were no hard restrictions imposed by construction cones before and after the detour—RD was a relaxing drive after all.

#### Eye tracking variables

**Figure 7:**
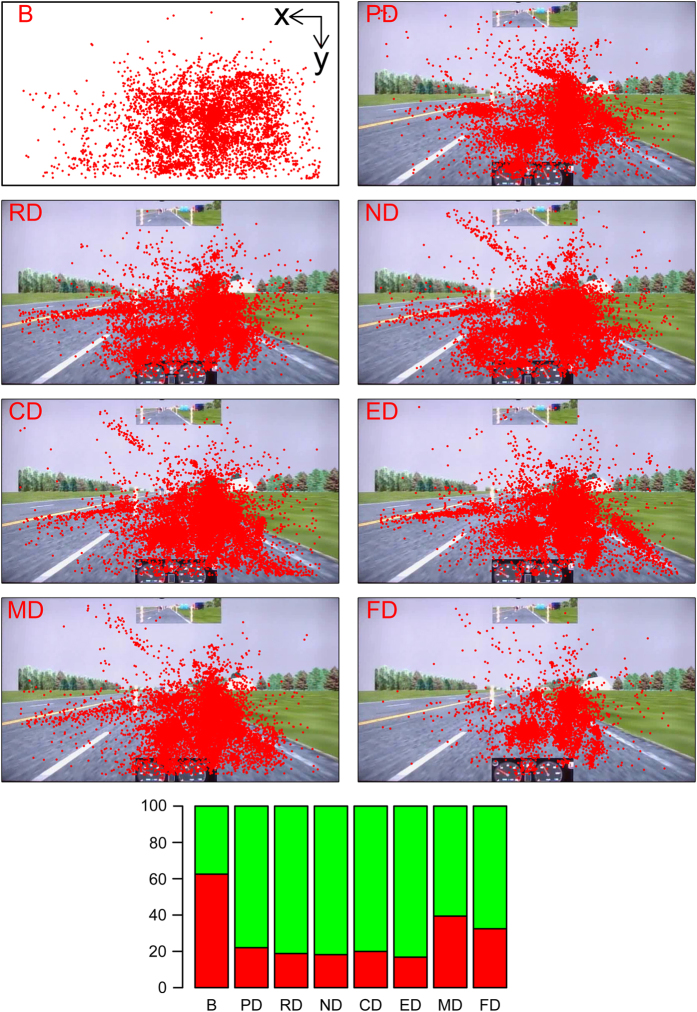
Top Eight Panels: Eye tracking scatterplots per experimental session, for all subjects. Bottom Panel: Bar plots indicating in red the percent of non-locking due to eyes off the screen (distraction) or eyes closed (sleeping/blinking).

The eye tracker recorded the x and y coordinates of the subject’s gaze at each point in time during the drives as well as during the baseline session. The top eight panels in Fig. 7 show the gaze scatterplots for all subjects per experimental session. In the drive panels PD, RD, CD, ED, MD, and FD, most of the gaze points are concentrated on the projection of the car’s itinerary. Gazing patterns are also notable at the bottom center of the simulator screen, where the speedometer is located, as well as on either side of the rear-view mirror at the top center of the simulator screen. In the baseline panel B, the scatterplot is more uniform, as the subjects were staring at a featureless screen.

To get an appreciation of the eye tracking scatterplots in the context of the experimental setting, detailed dimensions of the setup are given in Supplementary Fig. 1 of the manuscript’s Supplement. The origin and orientation of the eye tracking coordinate system is shown in panel B of Fig. 7.

The bar plot in the bottom panel of Fig. 7 shows in red the percent of gaze points per drive for which the eye tracker was unable to report values. This happened when the eye tracker could not lock on the subjects’ eyes because the subject was looking outside the simulator screen (e.g., texting) or had her/his eyes closed (e.g., sleeping or blinking). For the drives without any physical distractions, the missing eye tracking points are around 20%. For the MD and FD drives that feature physical distractions, the percentage of missing eye tracking points nearly doubles. Interestingly, the baseline session B features the highest percentage of missing eye tracking points. An investigation of the relevant facial videos reveals that many subjects fell asleep during the baseline session, and had their eyes closed for significant periods of time. Please note that these percentages apply to valid measurement sessions only (marked with ‘1’ in the ‘Dataset-Table-Index.xlsx’); they do not include missing measurement sessions (marked with ‘0’ in the ‘Dataset-Table-Index.xlsx’), where eye locking was not possible throughout the experimental session.

### Quality control of support media

The clocks of all the imaging and wearable sensing devices were synchronized at the beginning of each experimental day. Then, at the start of each experimental session, the experimenter struck a clapboard in front of the subject’s face. We used these clapboard scenes as the initial clipping points for the facial, thermal, and operational theater videos. All the frames in the recorded video streams had imprinted timestamps. We used the timestamps imprinted in the clapboard frames, to clip the corresponding physiological signals, synchronizing them to the start of the experimental sessions.

The end of each experimental driving session was marked by a stop sign that appeared in the middle of the simulator screen. We used the imprinted timestamps in the stop sign frames to clip the video streams and the corresponding physiological signals, synchronizing them to the end of the experimental sessions.

### Experimental validation

We used cognitive, emotional, and sensorimotor distractions to induce excessive stress during two phases in the CD, ED, and MD drives, respectively. Furthermore, we used four physiological variables—i.e., perinasal EDA, palm EDA, heart rate, breathing rate—to quantify peripheral manifestations of stress. It is fundamental to the validity of our dataset that indeed excessive stress was induced during the CD, ED, and MD drives, and that at least some of the chosen physiological variables successfully tracked it.

Regarding the first point, we demonstrated in ref. 7 that the experiment’s stressful loaded drives were perceived as such, by running a mixed effects model with dependent variables the NASA TLX sub-scales, fixed effects the different types of stressful loaded drives, and keeping the loaded drive with no stressors (ND) as the intercept.

Regarding the second point, we documented in ref. 7 that the perinasal EDA channel recorded significantly elevated sympathetic responses during the stressful phases P2 and P4 of the loaded drives (LD_*C*_ ≡ CD, LD_*E*_ ≡ ED, LD_*M*_ ≡ MD) with respect to the corresponding phases in the non-stressful loaded drive (LD_* Ø*_ ≡ ND), as expected. Here, we briefly recite the perinasal EDA test results from^7^, for completeness. Then, we apply the same tests to the other three physiological variables, presenting for them a more detailed analysis.

It is important to note that we did hypothesis testing against a two-tail alternative, setting levels of significance at *α*=0.0125 designated by **, or *α*=0.001 designated by ***. The *α*=0.0125 is Bonferroni-corrected for *n*=4 comparisons, referring to the four variables we used to characterize the stress states of drivers: perinasal EDA, palm EDA, heart rate, and breathing rate.

#### Perinasal EDA as a stress tracker

**Figure 8:**
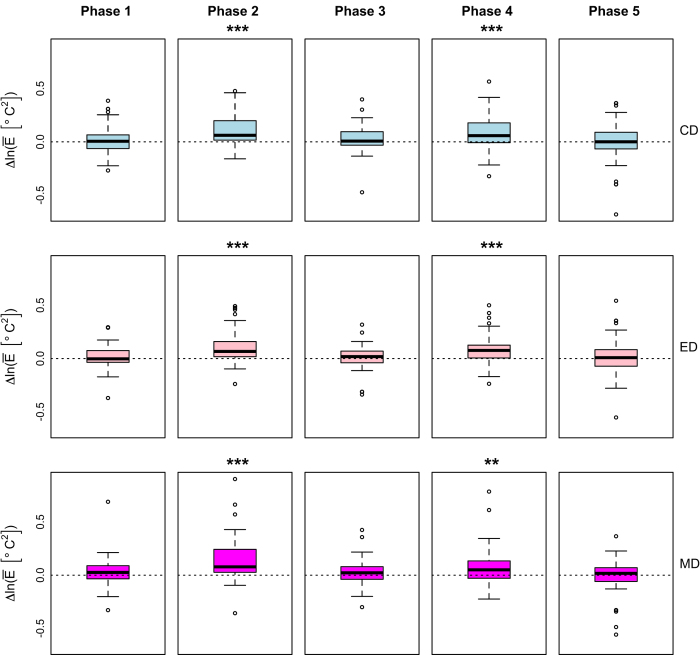
Validation of perinasal EDA channel. For more details see ref. 7.

For the subset of *n*=59 subjects for whom the extraction of the perinasal EDA signal was feasible, we computed for each driving phase P_*i*_ the distributions of paired differences between:

- Mean perinasal EDA in LD_*C*_ and LD_*Ø*_. This produced the first row of boxplots in Fig. 8, suggesting that cognitive distraction of subjects in phases P2_**LD**_*C*__ and P4_**LD**_*C*__ had as a result significant elevation of their mean perinasal EDA, with respect to phases P2_**LD**_*Ø*__ and P4_**LD**_*Ø*__ in the no-stressor drive (*P* < 0.001, paired *t*-tests in both cases).
- Mean perinasal EDA in LD_*E*_ and LD_*Ø*_. This produced the second row of boxplots in Fig. 8, suggesting that emotional distraction of subjects in phases $P2_{LD_{E}}$ and $P4_{LD_{E}}$ had as a result significant elevation of their mean perinasal EDA, with respect to phases P2_**LD**_*Ø*__ and P4_**LD**_*Ø*__ in the no-stressor drive (*P* < 0.001, paired *t*-tests in both cases).
- Mean perinasal EDA in LD_*M*_ and LD_*Ø*_. This produced the third row of boxplots in Fig. 8, suggesting that sensorimotor distraction of subjects in phases $P2_{LD_{M}}$ and $P4_{LD_{M}}$ had as a result significant elevation of their mean perinasal EDA, with respect to phases P2_**LD**_*Ø*__ and P4_**LD**_*Ø*__ in the no-stressor drive (*P*<0.001 in $P2_{LD_{M}}$ and *P*<0.0125 in $P4_{LD_{M}}$, paired *t*-tests in both cases).

Hence, perinasal EDA appears to reliably track sympathetic changes produced by both esoteric and physical stressors.

#### Palm EDA as a stress tracker

For the working set of *n*=68 subjects, we computed for each driving phase P_*i*_ the distributions of paired differences between:

- Mean palm EDA in LD_*C*_ and LD_*Ø*_ (equation (1))
  (1)S¯(⋅,C,Pi)=S¯(⋅,LDC,Pi)[kΩ]−S¯(⋅,LDO/,Pi)[kΩ]
- Mean palm EDA in LD_*E*_ and LD_*Ø*_ (equation (2))
  (2)S¯(⋅,E,Pi)=S¯(⋅,LDE,Pi)[kΩ]−S¯(⋅,LDO/,Pi)[kΩ]
- Mean palm EDA in LD_*M*_ and LD_*Ø*_ (equation (3))
  (3)S¯(⋅,M,Pi)=S¯(⋅,LDM,Pi)[kΩ]−S¯(⋅,LDO/,Pi)[kΩ]

**Figure 9:**
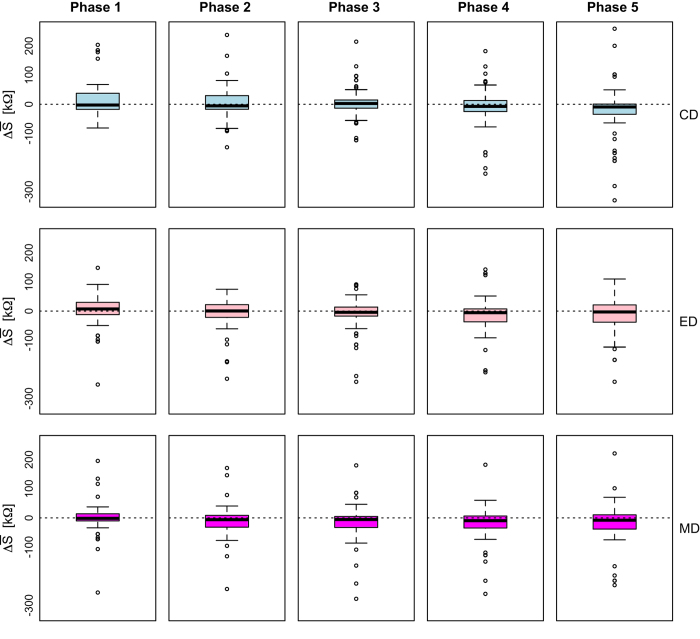
Validation of palm EDA channel.

Equation (1) produced the first row of boxplots in Fig. 9, suggesting that cognitive distraction of subjects in phases $P2_{LD_{C}}$ and $P4_{LD_{C}}$ had no significant effect on their mean palm EDA, with respect to phases $P2_{LD_{O\;/}}$ and $P4_{LD_{O\;/}}$ in the no-stressor drive (*P* > 0.05, paired *t*-tests in both cases).

Equation (2) produced the second row of boxplots in Fig. 9, suggesting that emotional distraction of subjects in phases $P2_{LD_{E}}$ and $P4_{LD_{E}}$ had no significant effect on their mean palm EDA, with respect to phases $P2_{LD_{O\;/}}$ and $P4_{LD_{O\;/}}$ in the no-stressor drive (*P* >0.05, paired *t*-tests in both cases).

Equation (3) produced the third row of boxplots in Fig. 9, suggesting that sensorimotor distraction of subjects in phases $P2_{LD_{M}}$ and $P4_{LD_{M}}$ had no significant effect on their mean palm EDA, with respect to phases $P2_{LD_{\emptyset }}$ and $P4_{LD_{\emptyset }}$ in the no-stressor drive (*P* >0.05, paired *t*-tests in both cases).

Hence, the palm EDA channel appears to be non-informative regarding sympathetic changes produced by either esoteric or physical stressors. In the context of the psychophysiological literature at large, this is surprising, as the palm EDA channel is one of the most reliable peripheral tracker of stress responses^19^. In the context of this application, however, such a disappointing performance is likely attributable to the constant friction of the sensor, as it is squeezed between the subject’s palm and the steering wheel.

#### Breathing rate as a stress tracker

For the working set of *n*=68 subjects, we computed for each driving phase P_*i*_ the distributions of paired differences between:

Mean breathing rate in LD_*C*_ and LD_*Ø*_ (equation (4))

(4)B¯(⋅,C,Pi)=B¯(⋅,LDC,Pi)[bpm]−B¯(⋅,LDO/,Pi)[bpm]

Mean breathing rate in LD_*E*_ and LD_*Ø*_ (equation (5))

(5)B¯(⋅,E,Pi)=B¯(⋅,LDE,Pi)[bpm]−B¯(⋅,LDO/,Pi)[bpm]

Mean breathing rate in LD_*M*_ and LD_*Ø*_ (equation (6))

(6)B¯(⋅,M,Pi)=B¯(⋅,LDM,Pi)[bpm]−B¯(⋅,LDO/,Pi)[bpm]

**Figure 10:**
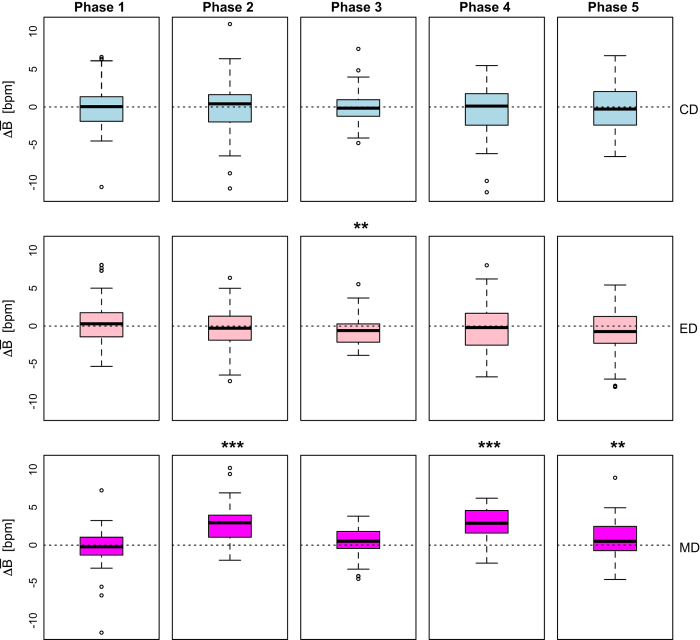
Validation of breathing rate channel.

Equation (4) produced the first row of boxplots in Fig. 10, suggesting that cognitive distraction of subjects in phases $P2_{LD_{C}}$ and $P4_{LD_{C}}$ had no significant effect on their mean breathing rate, with respect to phases $P2_{LD_{O\;/}}$ and $P4_{LD_{O\;/}}$ in the no-stressor drive (*P* > 0.05, paired *t*-tests in both cases).

Equation (5) produced the second row of boxplots in Fig. 10, suggesting that emotional distraction of subjects in phases $P2_{LD_{E}}$ and $P4_{LD_{E}}$ had no significant effect on their mean breathing rate, with respect to phases $P2_{LD_{O\;/}}$ and $P4_{LD_{O\;/}}$ in the no-stressor drive (*P* >0.05, paired *t*-tests in both cases).

Equation (6) produced the third row of boxplots in Fig. 10, suggesting that sensorimotor distraction of subjects in phases $P2_{LD_{M}}$ and $P4_{LD_{M}}$ had as a result significant elevation of their mean breathing rate, with respect to phases $P2_{LD_{O\;/}}$ and $P4_{LD_{O\;/}}$ in the no-stressor drive (*P* > 0.001, paired *t*-tests in both cases).

Hence, the breathing rate channel reliably tracks sympathetic changes only in the case of physically distracting stressors. The breathing rate channel is non-informative when it comes to sympathetic changes precipitated by moderate esoteric stressors, such as the cognitive and emotional stressors used in this experiment.

### Heart rate as a stress tracker

For the working set of *n*=68 subjects, we computed for each driving phase P_*i*_ the distributions of paired differences between:

- Mean heart rate in LD_*C*_ and LD_*Ø*_ (equation (7))
  (7)H¯(⋅,C,Pi)=H¯(⋅,LDC,Pi)[bpm]−H¯(⋅,LDO/,Pi)[bpm]
- Mean heart rate in LD_*E*_ and LD_*Ø*_ (equation (8))
  (8)H¯(⋅,E,Pi)=H¯(⋅,LDE,Pi)[bpm]−H¯(⋅,LDO/,Pi)[bpm]
- Mean heart rate in LD_*M*_ and LD_*Ø*_ (equation (9))
  (9)H¯(⋅,M,Pi))=H¯(⋅,LDM,Pi)[bpm]−H¯(⋅,LDO/,Pi)[bpm]

**Figure 11:**
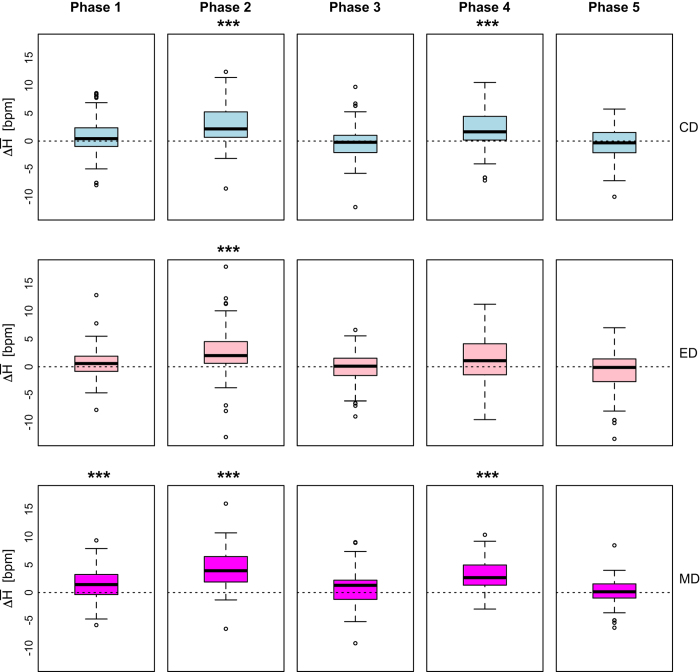
Validation of heart rate channel.

Equation (7) produced the first row of boxplots in Fig. 11, suggesting that cognitive distraction of subjects in phases $P2_{LD_{C}}$ and $P4_{LD_{C}}$ had as a result significant elevation of their mean heart rate, with respect to phases $P2_{LD_{O\;/}}$ and $P4_{LD_{O\;/}}$ in the no-stressor drive (*P*<0.001, paired *t*-tests in both cases).

Equation (8) produced the second row of boxplots in Fig. 11, suggesting that emotional distraction of subjects in phase $P2_{LD_{E}}$ had as a result significant elevation of their mean heart rate, with respect to phase $P2_{LD_{\emptyset }}$ in the no-stressor drive (*P*<0.001, paired *t*-test); by contrast, emotional distraction of subjects in phase $P4_{LD_{E}}$ had no significant effect on their mean heart rate, with respect to phase $P4_{LD_{\emptyset }}$ in the no-stressor drive (*P*>0.05, paired *t*-test).

Equation (9) produced the third row of boxplots in Fig. 11, suggesting that sensorimotor distraction of subjects in phases $P2_{LD_{M}}$ and $P4_{LD_{M}}$ had as a result significant elevation of their mean heart rate, with respect to phases $P2_{LD_{O\;/}}$ and $P4_{LD_{O\;/}}$ in the no-stressor drive (*P*<0.001, paired *t*-tests in both cases).

Hence, the heart rate channel appears to be almost as reliable a sympathetic tracker as the perinasal EDA channel, and in practice, these two channels can be used interchangeably in driving contexts. Investigators that plan to perform further research on this dataset are advised to use as primary explanatory variables perinasal EDA and heart rate.

## Usage Notes

The dataset includes both the thermal sequences (primary data), and the perinasal EDA signals that were extracted from these sequences. If one is interested to extract the perinasal signals anew, s/he has to use the S-Interface software^17^, selecting the region of interest (ROI) in the first frame of the radiometric sequence (.dat file) for each experimental session. The perinasal ROI is bound at the top by the subject’s nostrils, at the bottom by the subject’s lips, and on the left and right sides by the ends of the subject’s mouth. Based on this initial selection, the tracker is capable of following up this tissue area for the duration of the session, giving the chance to the physiological signal extractor to operate on a valid data set. In rare instances, the tracker momentarily fails. This happens when, for example, the subject performs a very abrupt head turn. The end result of such momentary failures are spikes in the perinasal signal. These spikes are removed by applying a noise-reduction algorithm reported in ref. 20. This algorithm is included in the S-Interface configuration. In even rarer instances, the tracker drifts away from the original ROI. This typically happens when the subject has turned her/his head at an extreme angle and stayed there for some time. The user can reposition the tracker by simply clicking the mouse in the perinasal area. The tracker is restored and the signal extraction process resumes from that point onward on the right footing.

The entire dataset can be visualized through Subject Book. Subject Book is a data management and visualization tool [http://subjectbook.times.uh.edu], which can be used with any dataset that follows its data structuring conventions. The Subject Book site for the current data set (SIMULATOR STUDY 1) can be viewed at [Data Citation 2]. Subject Book features three levels of abstraction: (a) The detailed level where all the data are visualized per subject, per session. (b) The subject level, where key explanatory and response variables are summarized in a subject ID format. (c) The study level, where a visual matrix conveys the results of the statistical tests on the study’s hypotheses.

## Additional Information

**How to cite this article:** Taamneh, S. *et al.* A multimodal dataset for various forms of distracted driving. *Sci. Data* 4:170110 doi: 10.1038/sdata.2017.110 (2017).

**Publisher’s note:** Springer Nature remains neutral with regard to jurisdictional claims in published maps and institutional affiliations.

## Supplementary Material



Supplementary Information

## References

[d1] Open Science Framework Taamneh S. 2016 https://doi.org/10.17605/OSF.IO/C42CN

[d2] Subject Book—SIMULATOR STUDY 1 Taamneh S. Pavlidis I. 2016 http://subjectbook.times.uh.edu/showPyramid?studyNo=23

