# Supplementary material for: A multimodal dataset for various forms of distracted driving
Source: Sci Data. 2017 Aug 15;4:170110. doi: 10.1038/sdata.2017.110 (PMC5827115; doi:10.1038/sdata.2017.110)
Supplement: Supplementary Information [file sdata2017110-s2.pdf]

A multimodal dataset for various forms of distracted driving

Salah Taamneh, Panagiotis Tsiamyrtzis, Malcolm Dcosta, Pradeep Buddharaju, Ashik Khatri,

Michael Manser, Thomas Ferris, Robert Wunderlich, Ioannis Pavlidis

Supplementary Material

Supplementary Figures

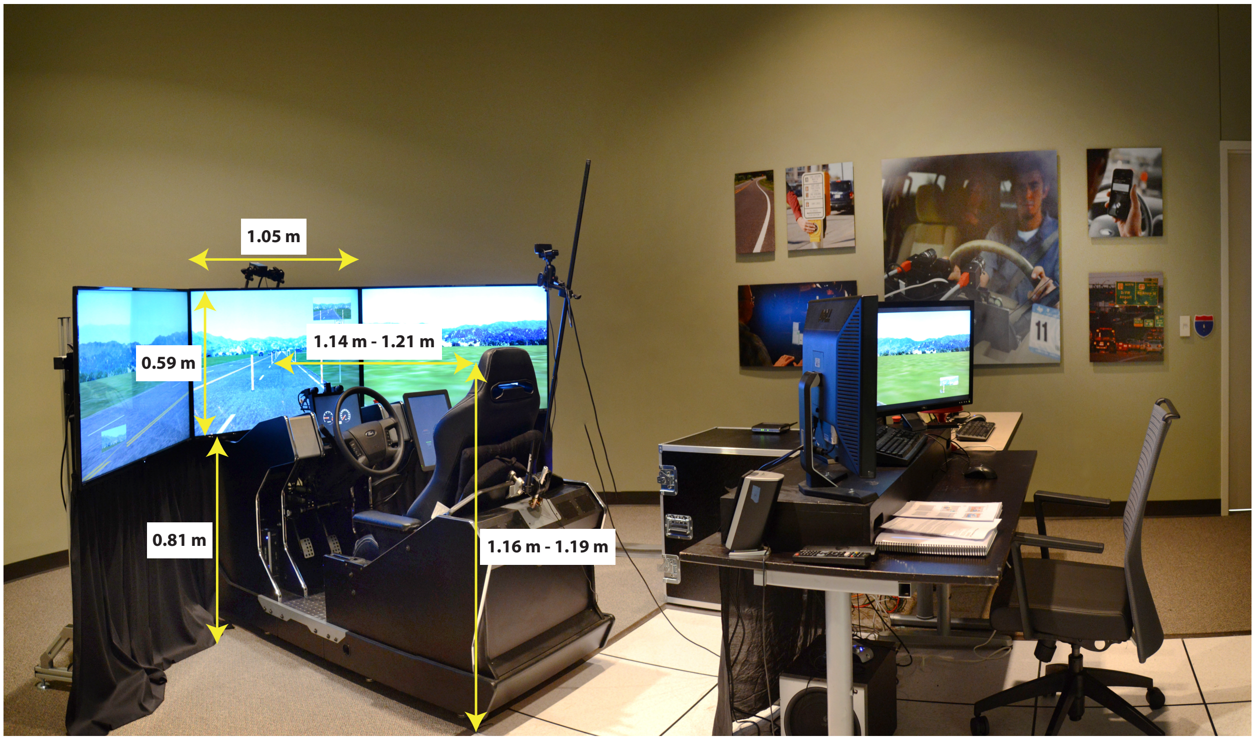

Supplement - Figure S1: Experimental setup annotated with distances. The distances are especially useful in analyzing the eye tracking data.

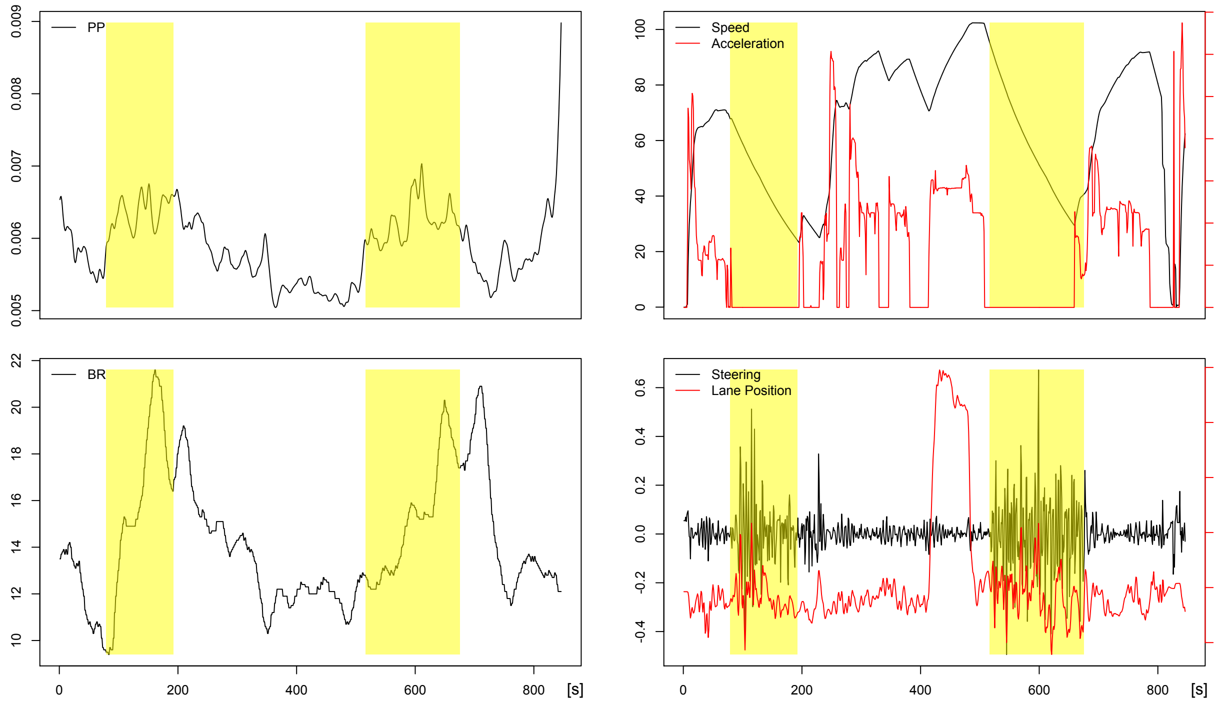

Supplement - Figure S2: Subject T029 - Sensorimotor Drive (MD). **Left Column:** Perinasal EDA (pp) and Breathing Rate (BR) signals - the physiological excitation during the two stressful phases is apparent. **Right Column:** Speed/Acceleration and Steering/Lane Position duets - the driving performance effects during the two stressful phases are apparent.

The dataset resides at:

https://osf.io/c42cn/

The dataset's visualization resides at:

http://subjectbook.times.uh.edu/showPyramid?studyNo=23

Dataset’s Index Table

Subject	Session	thermal data	pp	peda	HR	BR	performance (res)	stm	FACS	facial (avi1)	ROI (avi2)	OT (avi3)	eye	ROI CONFLICT?
T001	BL	1	1	NA	NA	NA	NA	NA	1	1	1	1	1	
T001	CD	1	1	1	-1	1	1	1	1	1	1	1	1	
T001	ED	1	1	1	-1	1	1	1	1	1	1	1	1	
T001	FDN	1	1	1	-1	1	1	1	1	1	1	1	1	
T001	MD	1	1	1	-1	1	1	1	1	1	1	1	1	
T001	ND	1	1	1	-1	1	1	NA	1	1	1	1	1	
T001	PD	1	1	1	-1	1	1	NA	1	1	1	1	1	
T001	RD	1	1	1	1	1	1	NA	1	1	1	1	1	
T002	BL	1	0	NA	NA	NA	NA	NA	0	0	0	0	0	
T002	CD	1	1	1	1	1	1	1	1	1	1	1	0	
T002	ED	1	1	1	1	1	1	1	1	1	1	1	0	
T002	FDL	1	1	1	1	1	0	1	1	1	1	1	0	
T002	MD	1	1	1	1	1	1	1	1	1	1	1	0	
T002	ND	1	1	1	1	1	1	NA	1	1	1	1	0	
T002	PD	1	1	1	1	1	1	NA	1	1	1	1	0	
T002	RD	1	1	1	1	1	1	NA	1	1	1	1	0	
T003	BL	1	1	NA	NA	NA	NA	NA	1	1	1	1	1	
T003	CD	1	1	1	1	1	1	1	1	1	1	1	1	
T003	ED	1	1	1	1	1	1	1	1	1	1	1	1	
T003	FDN	1	1	0	1	1	1	1	1	1	1	1	0	
T003	MD	1	1	1	1	1	1	1	1	1	1	1	0	
T003	ND	1	1	1	1	1	1	NA	1	1	1	1	1	
T003	PD	1	1	1	1	1	1	NA	1	1	1	1	1	
T003	RD	1	1	1	1	1	1	NA	1	1	1	1	1	
T004	BL	1	1	NA	NA	NA	NA	NA	1	1	1	1	1	
T004	CD	1	1	0	1	1	1	1	1	1	1	1	1	
T004	ED	1	1	0	1	1	1	1	1	1	1	1	1	
T004	FDL	1	1	0	1	1	1	1	1	1	1	1	1	
T004	MD	1	1	0	1	1	1	1	1	1	1	1	1	
T004	ND	1	1	1	1	1	1	NA	1	1	1	1	1	
T004	PD	1	1	1	1	1	1	NA	1	1	1	1	1	
T004	RD	1	1	1	1	1	1	NA	1	1	1	1	1	
T005	BL	1	1	NA	NA	NA	NA	NA	1	1	1	1	1	
T005	CD	1	1	1	1	1	1	1	1	1	1	1	1	
T005	ED	1	1	1	1	1	1	1	1	1	1	1	1	
T005	FDN	1	1	1	1	1	1	1	1	1	1	1	1	
T005	MD	1	1	1	1	1	1	1	1	1	1	1	1	
T005	ND	1	1	1	1	1	1	NA	1	1	1	1	1	
T005	PD	1	1	1	1	1	1	NA	1	1	1	1	1	
T005	RD	1	1	1	1	1	1	NA	1	1	1	1	1	
T006	BL	1	1	NA	NA	NA	NA	NA	1	1	1	1	1	
T006	CD	1	1	1	1	1	1	1	1	1	1	1	1	
T006	ED	1	1	1	1	1	1	1	1	1	1	1	1	
T006	FDL	1	1	1	1	1	1	1	1	1	1	1	1	
T006	MD	1	1	1	1	1	1	1	1	1	1	1	1	
T006	ND	1	1	1	-1	1	1	NA	1	1	1	1	1	
T006	PD	1	1	1	1	1	1	NA	1	1	1	1	1	
T006	RD	1	1	1	-1	1	1	NA	1	1	1	1	1	
T007	BL	1	N	NA	NA	NA	NA	NA	1	1	N	1	1	
T007	CD	1	N	1	1	1	1	1	1	1	N	1	0	
T007	ED	1	N	1	1	1	1	1	1	1	N	1	0	
T007	FDN	1	N	1	1	1	1	1	1	1	N	1	0	
T007	MD	1	N	1	1	1	1	1	1	1	N	1	0	
T007	ND	1	N	1	1	1	1	NA	1	1	N	1	0	
T007	PD	1	N	1	1	1	1	NA	1	1	N	1	0	
T007	RD	1	N	1	1	1	1	NA	1	1	N	1	0	
T008	BL	1	1	NA	NA	NA	NA	NA	1	1	1	1	1	
T008	CD	1	1	1	1	1	1	1	1	1	1	1	1	
T008	ED	1	1	1	1	1	1	1	1	1	1	0	0	
T008	FDL	1	1	1	1	1	1	1	1	1	1	1	0	
T008	MD	1	1	1	1	1	1	1	1	1	1	0	0	
T008	ND	1	1	1	1	1	1	NA	1	1	1	0	1	
T008	PD	1	1	-1	1	1	1	NA	1	1	1	1	1	
T008	RD	1	1	1	1	1	1	NA	1	1	1	1	1	
T009	BL	1	0	NA	NA	NA	NA	NA	0	0	0	0	0	
T009	CD	1	1	1	-1	1	1	1	1	1	1	1	1	
T009	ED	1	1	1	-1	1	1	1	1	1	1	1	1	
T009	FDN	1	1	1	-1	1	1	1	1	1	1	1	1	
T009	MD	1	1	1	-1	1	1	1	1	1	1	1	1	
T009	ND	1	1	1	-1	1	1	NA	1	1	1	1	1	
T009	PD	1	1	1	-1	1	1	NA	1	1	1	1	1	
T009	RD	1	1	1	-1	1	1	NA	1	1	1	1	1	
T010	BL	1	1	NA	NA	NA	NA	NA	1	1	1	1	1	
T010	CD	1	1	1	1	1	1	1	1	1	1	1	1	
T010	ED	1	1	1	1	1	1	1	1	1	1	1	1	
T010	FDL	1	1	1	1	1	1	1	1	1	1	1	1	
T010	MD	1	1	1	1	1	1	1	1	1	1	1	1	
T010	ND	1	1	1	1	1	1	NA	1	1	1	1	1	
T010	PD	1	1	-1	1	1	1	NA	1	1	1	1	1	
T010	RD	1	1	-1	1	1	1	NA	1	1	1	1	1	
T011	BL	1	1	NA	NA	NA	NA	NA	1	1	1	1	1	
T011	CD	1	1	0	1	1	1	1	1	1	1	1	1	

T011	ED	1	1	0	1	1	1	1	1	1	1	1	1
T011	FDN	1	1	0	1	1	1	1	1	1	1	1	1
T011	MD	1	1	0	1	1	1	1	1	1	1	1	1
T011	ND	1	1	0	1	1	1	NA	1	1	1	1	1
T011	PD	1	1	0	1	1	1	NA	1	1	1	1	1
T011	RD	1	1	0	1	1	1	NA	1	1	1	1	1
T012	BL	1	1	NA	NA	NA	NA	NA	1	1	1	1	1
T012	CD	1	1	1	-1	1	1	1	1	1	1	1	1
T012	ED	1	1	1	-1	1	1	1	1	1	1	1	1
T012	FDL	1	1	1	-1	1	1	1	1	1	1	1	1
T012	MD	1	1	1	-1	1	1	1	1	1	1	1	1
T012	ND	1	1	1	-1	1	1	NA	1	1	1	1	1
T012	PD	1	1	1	-1	1	1	NA	1	1	1	1	1
T012	RD	1	1	1	-1	1	1	NA	1	1	1	1	1
T013	BL	1	1	NA	NA	NA	NA	NA	1	1	1	1	1
T013	CD	1	1	0	1	1	1	1	1	1	1	1	1
T013	ED	1	1	0	1	1	1	1	1	1	1	1	1
T013	FDN	1	1	0	1	1	1	1	1	1	1	1	1
T013	MD	1	1	0	1	1	1	1	1	1	1	1	1
T013	ND	1	1	1	1	1	1	NA	1	1	1	1	1
T013	PD	1	1	1	1	1	1	NA	1	1	1	1	1
T013	RD	1	1	1	1	1	1	NA	1	1	1	1	1
T014	BL	1	1	NA	NA	NA	NA	NA	1	1	1	1	1
T014	CD	1	1	1	1	1	1	1	1	1	1	1	1
T014	ED	1	1	1	1	1	1	1	1	1	1	1	1
T014	FDL	1	1	1	1	1	1	1	1	1	1	1	1
T014	MD	1	1	1	1	1	1	1	1	1	1	1	1
T014	ND	1	1	1	1	1	1	NA	1	1	1	1	1
T014	PD	1	1	1	1	1	1	NA	1	1	1	1	1
T014	RD	1	1	1	1	1	1	NA	1	1	1	1	1
T015	BL	1	N	NA	NA	NA	NA	NA	1	1	N	1	0
T015	CD	1	N	1	-1	1	1	1	1	1	N	1	0
T015	ED	1	N	1	-1	1	1	1	1	1	N	0	0
T015	FDN	1	N	1	-1	1	1	1	1	1	N	1	0
T015	MD	1	N	1	-1	1	1	1	1	1	N	1	0
T015	ND	1	N	1	-1	1	1	NA	1	1	N	1	0
T015	PD	1	N	1	-1	1	1	NA	1	1	N	1	0
T015	RD	1	N	-1	-1	1	1	NA	1	1	N	1	1
T016	BL	1	1	NA	NA	NA	NA	NA	1	1	1	1	1
T016	CD	1	1	1	1	1	1	1	1	1	1	1	1
T016	ED	1	1	1	1	1	1	1	1	1	1	1	1
T016	FDL	1	1	1	1	1	1	1	1	1	1	1	1
T016	MD	1	1	1	1	1	1	1	1	1	1	1	1
T016	ND	1	1	1	1	1	1	NA	1	1	1	1	1
T016	PD	1	1	1	1	1	1	NA	1	1	1	1	1
T016	RD	1	1	1	1	1	1	NA	1	1	1	1	1
T017	BL	1	1	NA	NA	NA	NA	NA	1	1	1	0	0
T017	CD	1	1	1	1	1	1	1	1	1	1	1	0
T017	ED	1	1	1	1	1	1	1	1	1	1	1	0
T017	FDN	1	1	1	1	1	1	1	1	1	1	1	0
T017	MD	1	1	1	1	1	1	1	1	1	1	1	0
T017	ND	1	1	1	1	1	1	NA	1	1	1	1	0
T017	PD	1	1	1	1	1	1	NA	1	1	1	1	0
T017	RD	1	1	1	1	1	1	NA	1	1	1	1	0
T018	BL	1	1	NA	NA	NA	NA	NA	1	1	1	1	1
T018	CD	1	1	1	1	1	1	1	1	1	1	1	1
T018	ED	1	1	1	1	1	1	1	1	1	1	1	1
T018	FDL	1	1	1	1	1	1	1	1	1	1	1	1
T018	MD	1	1	1	1	1	1	1	1	1	1	1	1
T018	ND	1	1	1	1	1	1	NA	1	1	1	1	1
T018	PD	1	1	1	1	1	1	NA	1	1	1	1	1
T018	RD	1	1	1	1	1	1	NA	1	1	1	1	1
T019	BL	1	N	NA	NA	NA	NA	NA	1	1	N	1	1
T019	CD	1	N	1	1	1	1	1	1	1	N	1	1
T019	ED	1	N	1	1	1	1	1	1	1	N	1	1
T019	FDN	1	N	1	1	1	1	1	1	1	N	1	1
T019	MD	1	N	1	1	1	1	1	1	1	N	1	1
T019	ND	1	N	1	1	1	1	NA	1	1	N	1	1
T019	PD	1	N	1	1	1	1	NA	1	1	N	1	1
T019	RD	1	N	1	1	1	1	NA	1	1	N	1	1
T020	BL	1	1	NA	NA	NA	NA	NA	1	1	1	0	1
T020	CD	1	1	1	1	1	1	1	1	1	1	1	1
T020	ED	1	1	1	1	1	1	1	1	1	1	1	1
T020	FDL	1	1	1	1	1	1	1	1	1	1	1	1
T020	MD	1	1	1	1	1	1	1	1	1	1	1	1
T020	ND	1	1	1	1	1	1	NA	1	1	1	1	1
T020	PD	1	1	1	1	1	1	NA	1	1	1	1	1
T020	RD	1	1	1	1	1	1	NA	1	1	1	1	1
T021	BL	1	N	NA	NA	NA	NA	NA	1	1	N	1	1
T021	CD	1	N	1	1	1	1	1	1	1	N	1	1
T021	ED	1	N	-1	1	1	1	1	1	1	N	1	1
T021	FDN	1	N	-1	1	1	1	1	1	1	N	1	1
T021	MD	1	N	-1	1	1	1	1	1	1	N	1	1

T021	ND	1	N	1	1	1	1	NA	1	1	N	1	1
T021	PD	1	N	-1	1	1	1	NA	1	1	N	1	1
T021	RD	1	N	-1	1	1	1	NA	1	1	N	1	1
T022	BL	1	1	NA	NA	NA	NA	NA	1	1	1	0	1
T022	CD	1	1	1	1	1	1	1	1	1	1	1	1
T022	ED	1	1	1	1	1	1	1	1	1	1	1	1
T022	FDL	1	1	1	1	1	1	1	1	1	1	1	1
T022	MD	1	1	1	1	1	1	1	1	1	1	1	1
T022	ND	1	1	1	1	1	1	NA	1	1	1	1	1
T022	PD	1	1	-1	1	1	1	NA	1	1	1	1	1
T022	RD	1	1	-1	1	1	1	NA	1	1	1	1	1
T023	BL	1	1	NA	NA	NA	NA	NA	1	1	1	0	1
T023	CD	1	1	1	1	1	1	1	1	1	1	1	0
T023	ED	1	1	1	1	1	1	1	1	1	1	1	0
T023	FDN	1	1	1	1	1	1	1	1	1	1	1	0
T023	MD	1	1	1	1	1	1	1	1	1	1	1	0
T023	ND	1	1	1	1	1	1	NA	1	1	1	1	0
T023	PD	1	1	1	1	1	1	NA	1	1	1	1	0
T023	RD	1	1	1	1	1	1	NA	1	1	1	1	0
T024	BL	1	1	NA	NA	NA	NA	NA	1	1	1	0	1
T024	CD	1	1	1	1	1	1	1	1	1	1	1	1
T024	ED	1	1	1	1	1	1	1	1	1	1	1	1
T024	FDL	1	1	1	1	1	1	1	1	1	1	1	1
T024	MD	1	1	1	1	1	1	1	1	1	1	1	1
T024	ND	1	1	1	1	1	1	NA	1	1	1	1	1
T024	PD	1	1	1	1	1	1	NA	1	1	1	1	1
T024	RD	1	1	1	1	1	1	NA	1	1	1	1	1
T025	BL	1	1	NA	NA	NA	NA	NA	1	1	1	0	1
T025	CD	1	1	1	1	1	1	1	1	1	1	1	1
T025	ED	1	1	1	1	1	1	1	1	1	1	1	1
T025	FDL	1	1	-1	1	1	1	1	1	1	1	1	1
T025	MD	1	1	-1	1	1	1	1	1	1	1	1	1
T025	ND	1	1	1	1	1	1	NA	1	1	1	1	1
T025	PD	1	1	1	1	1	1	NA	1	1	1	1	0
T025	RD	1	1	1	1	1	1	NA	1	1	1	1	1
T026	BL	1	N	NA	NA	NA	NA	NA	1	1	N	1	1
T026	CD	1	N	1	1	1	1	1	1	1	N	1	1
T026	ED	1	N	0	1	1	1	1	1	1	N	1	0
T026	FDN	1	N	0	1	1	1	1	1	1	N	1	1
T026	MD	1	N	0	0	0	0	0	0	0	N	0	0
T026	ND	1	N	1	1	1	1	NA	1	1	N	1	1
T026	PD	1	N	1	1	1	1	NA	1	1	N	1	1
T026	RD	1	N	1	1	1	1	NA	1	1	N	1	1
T027	BL	1	1	NA	NA	NA	NA	NA	1	1	1	1	0
T027	CD	1	1	-1	1	1	1	1	1	1	1	1	0
T027	ED	1	1	-1	1	1	1	1	1	1	1	1	0
T027	FDL	1	1	0	1	1	1	1	1	1	1	1	0
T027	MD	1	1	0	1	1	1	1	1	1	1	1	0
T027	ND	1	1	-1	1	1	1	NA	1	1	1	1	0
T027	PD	1	1	-1	1	1	1	NA	1	1	1	1	0
T027	RD	1	1	-1	1	1	1	NA	1	1	1	1	0
T028	BL	1	1	NA	NA	NA	NA	NA	1	1	1	1	1
T028	CD	1	1	0	1	1	1	1	1	1	1	0	1
T028	ED	1	1	0	1	1	1	1	1	1	1	0	0
T028	FDN	1	1	0	1	1	1	1	1	1	1	0	0
T028	MD	1	1	0	1	1	1	0	1	1	1	0	0
T028	ND	1	1	0	1	1	1	NA	1	1	1	1	1
T028	PD	1	1	0	1	1	1	NA	1	1	1	0	1
T028	RD	1	1	0	1	1	1	NA	1	1	1	1	1
T029	BL	1	1	NA	NA	NA	NA	NA	1	1	1	0	0
T029	CD	1	1	1	1	1	1	1	1	1	1	1	0
T029	ED	1	1	1	1	1	1	1	1	1	1	1	0
T029	FDL	1	1	1	1	1	1	1	1	1	1	1	0
T029	MD	1	1	1	1	1	1	1	1	1	1	1	0
T029	ND	1	1	1	1	1	1	NA	1	1	1	1	0
T029	PD	1	1	1	1	1	1	NA	1	1	1	1	0
T029	RD	1	1	1	1	1	1	NA	1	1	1	1	0
T031	BL	1	1	NA	NA	NA	NA	NA	1	1	1	1	1
T031	CD	1	1	1	1	1	1	1	1	1	1	1	1
T031	ED	1	1	1	1	1	1	1	1	1	1	1	1
T031	FDL	1	1	1	1	1	1	1	1	1	1	1	1
T031	MD	1	1	1	1	1	1	1	1	1	1	1	1
T031	ND	1	1	1	1	1	1	NA	1	1	1	1	1
T031	PD	1	1	1	1	1	1	NA	1	1	1	1	1
T031	RD	1	1	1	1	1	1	NA	1	1	1	1	1
T032	BL	1	N	NA	NA	NA	NA	NA	1	1	N	1	1
T032	CD	1	N	0	0	0	1	1	1	1	N	1	0
T032	ED	1	N	0	0	0	1	1	1	1	N	1	0
T032	FDN	1	N	0	0	0	1	1	1	1	N	1	0
T032	MD	1	N	0	0	0	1	1	1	1	N	1	0
T032	ND	1	N	0	0	0	1	NA	1	1	N	1	0
T032	PD	1	N	0	0	0	1	NA	1	1	N	1	1
T032	RD	1	N	0	0	0	1	NA	1	1	N	1	0

T033	BL	1	1	NA	NA	NA	NA	NA	1	1	1	1	0
T033	CD	1	1	1	1	1	1	1	1	1	1	1	1
T033	ED	1	1	1	1	1	1	1	1	1	1	1	1
T033	FDL	1	1	1	1	1	1	1	1	1	1	1	1
T033	MD	1	1	1	1	1	1	1	1	1	1	1	1
T033	ND	1	1	1	1	1	1	NA	1	1	1	1	1
T033	PD	1	1	1	1	1	1	NA	1	1	1	1	1
T033	RD	1	1	1	1	1	1	NA	1	1	1	1	1
T034	BL	1	1	NA	NA	NA	NA	NA	1	1	1	1	1
T034	CD	1	1	1	1	1	1	1	1	1	1	1	1
T034	ED	1	1	1	1	1	0	1	1	1	1	1	1
T034	FDN	1	1	1	1	1	0	1	1	1	1	1	1
T034	MD	1	1	1	1	1	1	1	1	1	1	1	1
T034	ND	1	1	1	1	1	1	NA	1	1	1	1	1
T034	PD	1	1	1	1	1	1	NA	1	1	1	1	1
T034	RD	1	1	1	1	1	0	NA	1	1	1	1	1
T035	BL	1	1	NA	NA	NA	NA	NA	1	1	1	1	1
T035	CD	1	1	-1	1	1	1	1	1	1	1	1	1
T035	ED	1	1	-1	1	1	1	1	1	1	1	1	1
T035	FDL	1	1	-1	1	1	1	1	1	1	1	1	1
T035	MD	1	1	-1	1	1	1	1	1	1	1	1	1
T035	ND	1	1	-1	1	1	1	NA	1	1	1	1	1
T035	PD	1	1	-1	1	1	1	NA	1	1	1	1	1
T035	RD	1	1	-1	1	1	1	NA	1	1	1	1	1
T036	BL	1	1	NA	NA	NA	NA	NA	1	1	1	0	0
T036	CD	1	1	1	1	1	1	1	1	1	1	1	0
T036	ED	1	1	1	1	1	1	1	1	1	1	1	0
T036	FDN	1	1	1	1	1	1	1	1	1	1	1	0
T036	MD	1	1	1	1	1	1	1	1	1	1	1	0
T036	ND	1	1	1	1	1	1	NA	1	1	1	1	0
T036	PD	1	1	1	1	1	1	NA	1	1	1	1	0
T036	RD	1	1	1	1	1	1	NA	1	1	1	1	0
T038	BL	1	1	NA	NA	NA	NA	NA	1	1	1	0	1
T038	CD	1	1	1	1	1	1	1	1	1	1	0	0
T038	ED	1	1	1	1	1	1	1	1	1	1	0	0
T038	FDN	1	1	1	1	1	1	1	1	1	1	0	0
T038	MD	1	1	1	1	1	1	1	1	1	1	0	0
T038	ND	1	1	1	1	1	1	NA	1	1	1	0	0
T038	PD	1	1	1	1	1	1	NA	1	1	1	0	1
T038	RD	1	1	1	1	1	1	NA	1	1	1	0	0
T039	BL	1	1	NA	NA	NA	NA	NA	1	1	1	1	1
T039	CD	1	1	1	1	1	1	1	1	1	1	1	1
T039	ED	1	1	1	1	1	1	1	1	1	1	1	1
T039	FDL	1	1	1	1	1	1	1	1	1	1	1	1
T039	MD	1	1	1	1	1	1	1	1	1	1	1	1
T039	ND	1	1	1	1	1	1	NA	1	1	1	1	1
T039	PD	1	1	1	1	1	1	NA	0	1	1	1	1
T039	RD	1	1	1	1	1	1	NA	1	1	1	1	1
T040	BL	1	1	NA	NA	NA	NA	NA	1	1	1	1	1
T040	CD	1	1	0	1	1	1	1	1	1	1	1	1
T040	ED	1	1	0	1	1	1	1	1	1	1	1	1
T040	FDN	1	1	0	1	1	1	1	1	1	1	1	1
T040	MD	1	1	0	1	1	1	1	1	1	1	1	1
T040	ND	1	1	0	1	1	1	NA	1	1	1	1	1
T040	PD	1	1	0	1	1	1	NA	1	1	1	1	1
T040	RD	1	1	0	1	1	1	NA	1	1	1	1	1
T041	BL	1	1	NA	NA	NA	NA	NA	1	1	1	1	1
T041	CD	1	1	1	-1	1	1	1	1	1	1	1	1
T041	ED	1	1	1	-1	1	1	1	0	0	1	1	1
T041	FDL	1	1	1	-1	1	1	1	1	1	1	1	1
T041	MD	1	1	1	-1	1	1	1	1	1	1	1	1
T041	ND	1	1	-1	-1	1	1	NA	1	1	1	1	1
T041	PD	1	1	1	-1	1	1	NA	1	1	1	1	1
T041	RD	1	1	1	-1	1	1	NA	0	1	1	1	1
T042	BL	1	N	NA	NA	NA	NA	NA	1	1	N	1	0
T042	CD	1	N	1	1	1	1	1	1	1	N	1	0
T042	ED	1	N	1	1	1	1	1	1	1	N	1	0
T042	FDN	1	N	1	1	1	1	1	1	1	N	1	0
T042	MD	1	N	1	1	1	1	1	1	1	N	1	0
T042	ND	1	N	1	1	1	1	NA	1	1	N	1	0
T042	PD	1	N	-1	1	1	1	NA	1	1	N	1	0
T042	RD	1	N	-1	1	1	1	NA	1	1	N	1	0
T043	BL	1	0	NA	NA	NA	NA	NA	0	0	0	0	0
T043	CD	1	1	1	1	1	1	1	1	1	1	1	1
T043	ED	1	1	1	1	1	1	1	1	1	1	1	1
T043	FDL	1	1	1	1	1	1	1	1	1	1	1	1
T043	MD	1	1	1	1	1	1	1	1	1	1	1	1
T043	ND	1	1	1	1	1	1	NA	0	1	1	1	1
T043	PD	1	1	1	1	1	1	NA	1	1	1	1	1
T043	RD	1	1	1	1	1	1	NA	1	1	1	1	1
T044	BL	1	1	NA	NA	NA	NA	NA	1	1	1	1	1
T044	CD	1	1	1	1	1	1	1	1	1	1	1	1
T044	ED	1	1	1	1	1	1	1	1	1	1	1	1

T044	FDN	1	1	1	1	1	1	1	1	1	1	1	1
T044	MD	1	1	1	1	1	1	1	1	1	1	1	1
T044	ND	1	1	1	1	1	1	NA	1	1	1	1	1
T044	PD	1	1	1	1	1	1	NA	1	1	1	1	1
T044	RD	1	1	1	1	1	1	NA	1	1	1	1	1
T045	BL	1	1	NA	NA	NA	NA	NA	0	1	1	0	1
T045	CD	1	1	1	1	1	1	1	0	1	1	1	1
T045	ED	1	1	1	1	1	1	1	0	1	1	1	1
T045	FDL	1	1	1	1	1	1	1	0	1	1	1	1
T045	MD	1	1	1	1	1	1	1	0	1	1	1	1
T045	ND	1	1	1	1	1	1	NA	0	1	1	1	1
T045	PD	1	1	1	1	1	1	NA	0	1	1	1	0
T045	RD	1	1	1	1	1	1	NA	0	1	1	1	0
T046	BL	1	N	NA	NA	NA	NA	NA	1	IRB	N	IRB	1
T046	CD	1	N	1	1	1	1	1	1	IRB	N	IRB	1
T046	ED	1	N	1	1	1	1	1	1	IRB	N	IRB	1
T046	FDN	1	N	1	1	1	1	0	0	IRB	N	IRB	0
T046	MD	1	N	1	1	1	1	1	1	IRB	N	IRB	1
T046	ND	1	N	1	1	1	1	NA	1	IRB	N	IRB	1
T046	PD	1	N	1	1	1	1	NA	1	IRB	N	IRB	1
T046	RD	1	N	0	0	0	0	NA	0	IRB	N	IRB	0
T047	BL	1	1	NA	NA	NA	NA	NA	1	1	1	0	0
T047	CD	1	1	1	1	1	1	1	1	1	1	1	0
T047	ED	1	1	1	1	1	1	1	1	1	1	1	0
T047	FDL	1	1	1	1	1	1	1	1	1	1	1	0
T047	MD	1	1	1	1	1	1	1	1	1	1	1	0
T047	ND	1	1	1	1	1	1	NA	1	1	1	1	0
T047	PD	1	1	1	1	1	1	NA	1	1	1	1	0
T047	RD	1	1	1	1	1	1	NA	1	1	1	1	0
T050	BL	1	1	NA	NA	NA	NA	NA	1	1	1	0	1
T050	CD	1	1	-1	1	1	1	1	1	1	1	1	1
T050	ED	1	1	-1	1	1	1	1	0	1	1	1	1
T050	FDN	1	1	1	1	1	1	1	1	1	1	1	0
T050	MD	1	1	-1	1	1	1	1	1	1	1	1	1
T050	ND	1	1	-1	1	1	1	NA	0	1	1	1	1
T050	PD	1	1	0	1	1	1	NA	0	1	1	1	1
T050	RD	1	1	0	1	1	1	NA	1	1	1	1	0
T051	BL	1	N	NA	NA	NA	NA	NA	1	1	N	1	1
T051	CD	1	N	1	1	1	1	1	1	1	N	1	1
T051	ED	1	N	1	1	1	1	1	1	0	N	1	1
T051	FDN	1	N	1	1	1	1	0	1	1	N	1	1
T051	MD	1	N	1	1	1	1	1	0	1	N	1	1
T051	ND	1	N	1	1	1	1	NA	1	1	N	1	1
T051	PD	1	N	1	1	1	1	NA	1	1	N	1	1
T051	RD	1	N	1	1	1	1	NA	1	1	N	1	1
T054	BL	1	1	NA	NA	NA	NA	NA	1	1	1	0	1
T054	CD	1	1	-1	1	-1	1	1	1	1	1	1	1
T054	ED	1	1	1	1	1	1	1	1	1	1	1	1
T054	FDN	1	1	1	1	1	1	1	1	1	1	1	1
T054	MD	1	1	1	1	1	1	1	1	1	1	1	1
T054	ND	1	1	1	1	1	1	NA	1	1	1	1	1
T054	PD	1	1	1	1	1	1	NA	1	1	1	1	1
T054	RD	1	1	1	1	1	0	NA	1	1	1	1	1
T055	BL	1	1	NA	NA	NA	NA	NA	1	1	1	1	1
T055	CD	1	1	1	1	1	1	1	1	1	1	0	0
T055	ED	1	1	-1	1	1	1	1	1	1	1	0	0
T055	FDL	1	1	0	1	1	0	1	1	1	1	1	1
T055	MD	1	1	0	1	1	0	1	0	0	1	1	1
T055	ND	1	1	-1	1	1	1	NA	1	1	1	0	0
T055	PD	1	1	1	1	1	1	NA	1	1	1	0	0
T055	RD	1	1	-1	1	1	0	NA	1	1	1	0	0
T060	BL	1	1	NA	NA	NA	NA	NA	1	1	1	1	1
T060	CD	1	1	1	1	1	1	1	1	1	1	1	1
T060	ED	1	1	1	1	1	1	1	1	1	1	1	1
T060	FDN	1	1	1	1	1	1	1	1	1	1	1	1
T060	MD	1	1	1	1	1	1	1	1	1	1	1	1
T060	ND	1	1	1	1	1	1	NA	1	1	1	1	1
T060	PD	1	1	1	1	1	0	NA	1	1	1	1	1
T060	RD	1	1	1	1	1	1	NA	1	1	1	1	1
T061	BL	1	1	NA	NA	NA	NA	NA	1	1	1	1	1
T061	CD	1	1	1	1	1	1	1	1	1	1	1	1
T061	ED	1	1	1	1	1	1	1	1	1	1	1	1
T061	FDL	1	1	1	1	1	1	1	1	1	1	1	1
T061	MD	1	1	1	1	1	1	1	1	1	1	1	1
T061	ND	1	1	1	1	1	1	NA	1	1	1	1	1
T061	PD	1	1	0	0	0	0	NA	0	0	1	1	1
T061	RD	1	1	1	1	1	1	NA	1	1	1	1	1
T062	BL	1	1	NA	NA	NA	NA	NA	1	1	1	1	1
T062	CD	1	1	1	-1	1	1	1	1	1	1	1	1
T062	ED	1	1	1	1	1	1	1	1	1	1	1	1
T062	FDN	1	1	1	1	1	1	1	1	1	1	1	1
T062	MD	1	1	0	0	0	0	1	0	0	1	1	1
T062	ND	1	1	1	1	1	1	NA	1	1	1	1	1

T062	PD	1	1	1	-1	1	1	NA	1	1	1	1	1	
T062	RD	1	1	1	1	1	1	NA	1	1	1	1	1	
T064	BL	1	1	NA	NA	NA	NA	NA	1	1	1	1	1	
T064	CD	1	1	0	-1	1	1	1	1	1	1	1	1	
T064	ED	1	1	0	-1	1	1	1	1	1	1	1	1	
T064	FDN	1	1	0	-1	1	1	1	1	1	1	1	1	
T064	MD	1	1	0	1	1	1	1	1	1	1	1	1	
T064	ND	1	1	0	-1	1	1	NA	1	1	1	1	1	
T064	PD	1	1	0	1	1	1	NA	1	1	1	1	1	
T064	RD	1	1	0	1	1	1	NA	1	1	1	1	1	
T066	BL	1	1	NA	NA	NA	NA	NA	1	1	1	1	1	
T066	CD	1	1	1	1	1	1	1	1	1	1	1	1	
T066	ED	1	1	1	1	1	1	1	1	1	1	1	1	
T066	FDN	1	1	1	1	1	1	1	1	1	1	1	1	
T066	MD	1	1	1	1	1	1	1	1	1	1	1	1	
T066	ND	1	1	1	1	1	1	NA	1	1	1	1	1	
T066	PD	1	1	1	1	1	1	NA	1	1	1	1	1	
T066	RD	1	1	1	1	1	1	NA	1	1	1	1	1	
T068	BL	1	1	NA	NA	NA	NA	NA	0	1	1	1	1	
T068	CD	1	1	1	1	1	1	1	0	1	1	1	1	
T068	ED	1	1	1	1	1	1	1	0	1	1	1	1	
T068	FDL	1	1	1	1	1	1	1	0	1	1	1	1	
T068	MD	1	1	1	1	1	1	1	0	1	1	0	1	
T068	ND	1	1	1	1	1	1	NA	0	1	1	1	1	
T068	PD	1	1	1	1	1	0	NA	0	1	1	0	1	
T068	RD	1	1	1	1	1	1	NA	0	1	1	0	1	
T073	BL	1	1	NA	NA	NA	NA	NA	1	1	1	1	1	
T073	CD	1	1	-1	1	1	0	1	1	1	1	1	1	
T073	ED	1	1	-1	1	1	1	1	1	1	1	1	1	
T073	FDN	1	1	-1	1	1	1	1	1	0	1	1	1	
T073	MD	1	1	-1	1	1	1	1	1	1	1	1	1	
T073	ND	1	1	-1	1	1	0	NA	1	1	1	1	1	
T073	PD	1	1	-1	1	1	1	NA	1	1	1	1	1	
T073	RD	1	1	-1	1	1	0	NA	1	1	1	1	1	
T074	BL	1	1	NA	NA	NA	NA	NA	1	IRB	IRB	IRB	1	
T074	CD	1	1	-1	-1	1	1	1	1	IRB	IRB	IRB	1	
T074	ED	1	1	1	-1	1	1	1	1	IRB	IRB	IRB	1	
T074	FDL	1	1	1	-1	1	1	1	1	IRB	IRB	IRB	1	
T074	MD	1	1	-1	-1	1	1	1	1	IRB	IRB	IRB	1	
T074	ND	0	1	1	-1	1	1	NA	1	IRB	IRB	IRB	1	
T074	PD	1	1	-1	-1	1	1	NA	1	IRB	IRB	IRB	1	
T074	RD	1	1	1	-1	1	1	NA	1	IRB	IRB	IRB	1	
T075	BL	1	1	NA	NA	NA	NA	NA	1	1	1	1	1	
T075	CD	1	1	1	1	1	1	1	1	1	1	1	1	
T075	ED	1	1	-1	1	1	1	1	1	1	1	1	1	
T075	FDN	1	1	1	1	1	1	1	1	1	1	1	1	
T075	MD	1	0	0	0	0	0	0	0	0	0	0	0	
T075	ND	1	1	1	1	1	1	NA	1	1	1	1	1	
T075	PD	1	1	1	1	1	1	NA	1	1	1	1	1	
T075	RD	1	1	1	1	1	1	NA	1	1	0	1	0	ROI CONFLICT
T076	BL	1	1	NA	NA	NA	NA	NA	1	1	1	1	1	
T076	CD	1	1	-1	1	1	1	1	1	1	1	1	1	
T076	ED	1	1	-1	1	1	1	1	1	1	1	1	1	
T076	FDL	1	1	1	1	1	1	1	1	1	1	1	1	
T076	MD	1	1	1	1	1	1	1	1	1	1	1	1	
T076	ND	1	1	-1	1	1	1	NA	1	1	1	1	1	
T076	PD	1	1	-1	1	1	1	NA	1	1	1	1	0	
T076	RD	1	1	-1	1	1	1	NA	1	1	1	1	1	
T077	BL	1	1	NA	NA	NA	NA	NA	1	1	1	1	1	
T077	CD	1	1	1	1	1	1	1	1	1	1	1	1	
T077	ED	1	1	1	1	1	1	1	1	1	1	1	1	
T077	FDN	1	1	1	1	1	1	1	1	1	1	1	1	
T077	MD	1	1	1	1	1	1	1	1	1	1	1	1	
T077	ND	1	1	1	1	1	1	NA	1	1	1	1	1	
T077	PD	1	1	-1	1	1	1	NA	1	1	1	1	1	
T077	RD	1	1	1	1	1	1	NA	1	1	1	1	1	
T079	BL	1	1	NA	NA	NA	NA	NA	1	1	1	1	1	
T079	CD	1	1	1	1	1	1	1	1	1	1	1	1	
T079	ED	1	1	1	1	1	1	1	1	1	1	1	1	
T079	FDN	1	1	1	1	1	1	1	1	1	1	1	1	
T079	MD	1	1	-1	1	1	1	1	1	1	1	1	1	
T079	ND	1	1	1	1	1	1	NA	1	1	1	1	1	
T079	PD	1	1	1	1	1	1	NA	1	1	1	1	1	
T079	RD	1	1	1	1	1	1	NA	1	1	1	1	1	
T080	BL	1	1	NA	NA	NA	NA	NA	1	1	1	1	1	
T080	CD	1	1	1	1	1	1	1	1	1	1	1	1	
T080	ED	1	1	1	1	1	1	1	1	1	1	1	1	
T080	FDN	1	1	1	1	1	1	1	1	1	1	1	1	
T080	MD	1	1	1	1	1	1	1	1	1	1	1	1	
T080	ND	1	1	1	1	1	1	NA	1	1	1	1	1	
T080	PD	1	1	1	1	1	1	NA	1	1	1	1	1	
T080	RD	1	1	1	1	1	1	NA	1	1	1	1	1	
T081	BL	1	1	NA	NA	NA	NA	NA	1	1	1	1	1	

T081	CD	1	1	1	1	1	1	1	1	1	1	1	1	
T081	ED	1	1	1	1	1	1	1	1	1	1	1	1	
T081	FDL	1	1	1	1	1	1	1	1	1	1	1	1	
T081	MD	1	1	1	1	1	1	1	1	1	1	1	1	
T081	ND	1	1	1	1	1	1	NA	1	1	1	1	1	
T081	PD	1	1	1	1	1	1	NA	1	1	1	1	1	
T081	RD	1	1	1	1	1	1	NA	1	1	1	1	1	
T082	BL	1	1	NA	NA	NA	NA	NA	1	1	1	1	1	
T082	CD	1	1	0	1	1	1	1	1	1	1	1	1	
T082	ED	1	1	0	1	1	1	1	1	1	1	1	1	
T082	FDL	1	1	0	1	1	1	1	1	1	1	1	1	
T082	MD	1	1	0	1	1	1	1	1	1	1	1	1	
T082	ND	1	1	0	1	1	1	NA	1	1	1	1	1	
T082	PD	1	1	0	1	1	1	NA	1	1	1	1	1	
T082	RD	1	1	0	1	1	1	NA	1	1	1	1	1	
T083	BL	1	1	NA	NA	NA	NA	NA	1	1	1	1	1	
T083	CD	1	1	-1	1	1	1	1	1	1	1	1	1	
T083	ED	1	1	-1	1	1	1	1	1	1	1	1	1	
T083	FDL	1	1	1	1	1	1	1	1	1	0	1	1	ROI CONFLICT
T083	MD	1	1	-1	1	1	1	1	1	1	1	1	1	
T083	ND	1	1	-1	1	1	1	NA	1	1	1	1	1	
T083	PD	1	1	-1	1	1	1	NA	1	1	1	1	1	
T083	RD	1	1	-1	1	1	1	NA	1	1	1	1	1	
T084	BL	1	1	NA	NA	NA	NA	NA	1	1	1	1	1	
T084	CD	1	1	1	1	1	1	1	0	1	1	1	1	
T084	ED	1	1	1	1	1	1	1	1	1	1	1	1	
T084	FDL	1	1	1	1	1	1	1	1	1	0	1	1	ROI CONFLICT
T084	MD	1	1	1	1	1	1	1	1	1	1	1	1	
T084	ND	1	1	1	1	1	0	NA	1	1	1	1	1	
T084	PD	1	1	-1	1	1	1	NA	1	1	1	1	1	
T084	RD	1	1	1	1	1	1	NA	1	1	1	1	1	
T086	BL	1	1	NA	NA	NA	NA	NA	1	1	0	1	1	ROI CONFLICT
T086	CD	1	1	-1	1	1	1	1	1	1	1	1	1	
T086	ED	1	1	1	1	1	1	1	1	1	1	1	1	
T086	FDN	1	1	-1	1	1	1	1	1	1	1	1	1	
T086	MD	1	1	-1	1	1	1	1	1	1	1	1	1	
T086	ND	1	1	-1	1	1	1	NA	1	1	1	1	0	
T086	PD	1	1	-1	1	1	1	NA	1	1	1	1	1	
T086	RD	1	1	-1	1	1	1	NA	1	1	1	1	1	
T088	BL	1	1	NA	NA	NA	NA	NA	1	1	1	1	1	
T088	CD	1	1	0	0	0	1	1	1	1	1	1	1	
T088	ED	1	1	0	0	0	1	1	1	1	1	1	1	
T088	FDL	1	1	0	0	0	1	1	1	1	1	1	1	
T088	MD	1	1	0	0	0	1	1	1	1	1	1	1	
T088	ND	1	1	0	0	0	1	NA	1	1	1	1	1	
T088	PD	1	1	0	0	0	1	NA	1	1	1	1	1	
T088	RD	1	1	0	0	0	1	NA	1	1	1	1	1	
		**dat**	**pp**	**peda**	**hr**	**br**	**res2**	**stm**	**facs**	**avi1**	**avi2**	**avi3**	**eye**	
VALID		543	468	338	408	456	457	267	509	517	456	486	426	4905
INVALID		0	0	67	49	1	0	0	0	0	0	0	0	117
MISSING		1	4	71	19	19	19	5	35	11	8	42	118	234
NOT APPLICABLE				68	68	68	68	272						544
NASAI ONLY			72								72			144
IRB RESTRICTIONS										16	8	16		40
**SUMS**		**544**	**544**	**544**	**544**	**544**	**544**	**544**	**544**	**544**	**544**	**544**	**544**	
